# Oncogenic Gain of Function in Glioblastoma Is Linked to Mutant p53 Amyloid Oligomers

**DOI:** 10.1016/j.isci.2020.100820

**Published:** 2020-01-08

**Authors:** Murilo M. Pedrote, Michelle F. Motta, Giulia D.S. Ferretti, Douglas R. Norberto, Tania C.L.S. Spohr, Flavia R.S. Lima, Enrico Gratton, Jerson L. Silva, Guilherme A.P. de Oliveira

**Affiliations:** 1 Institute of Medical Biochemistry Leopoldo de Meis, National Institute of Science and Technology for Structural Biology and Bioimaging, National Center of Nuclear Magnetic Resonance Jiri Jonas, Federal University of Rio de Janeiro, Rio de Janeiro, Rio de Janeiro 21941-901, Brazil; 2 Universidade Federal do ABC, Centro de Ciências Naturais e Humanas. Av. dos Estados, 5001 Sta. Terezinha, Santo André, São Paulo 21941-590, Brazil; 3 Laboratório de Biomedicina do Cérebro, Instituto Estadual do Cérebro Paulo Niemeyer (IECPN), Secretaria de Estado de Saúde, Rio de Janeiro, Brazil; 4 Instituto de Ciências Biomédicas, Universidade Federal do Rio de Janeiro, Rio de Janeiro, Brazil; 5 Laboratory for Fluorescence Dynamics, Biomedical Engineering Department, University of California, Irvine, CA 92697-2717, USA; 6 Department of Biochemistry and Molecular Genetics, University of Virginia, Charlottesville, VA 22908-0733, USA

**Keywords:** Structural Biology, Protein Structure Aspects, Biophysics, Cancer

## Abstract

Tumor-associated p53 mutations endow cells with malignant phenotypes, including chemoresistance. Amyloid-like oligomers of mutant p53 transform this tumor suppressor into an oncogene. However, the composition and distribution of mutant p53 oligomers are unknown and the mechanism involved in the conversion is sparse. Here, we report accumulation of a p53 mutant within amyloid-like p53 oligomers in glioblastoma-derived cells presenting a chemoresistant gain-of-function phenotype. Statistical analysis from fluorescence fluctuation spectroscopy, pressure-induced measurements, and thioflavin T kinetics demonstrates the distribution of oligomers larger than the active tetrameric form of p53 in the nuclei of living cells and the destabilization of native-drifted p53 species that become amyloid. Collectively, these results provide insights into the role of amyloid-like mutant p53 oligomers in the chemoresistance phenotype of malignant and invasive brain tumors and shed light on therapeutic options to avert cancer.

## Highlights

• Amyloid oligomers transform p53 tumor suppressor into an oncogene
• Amyloid-like mutant p53 oligomers occur in chemoresistant glioblastoma cells
• p53 oligomer larger than tetramers is detected in the nuclei of living cells
• Gain-of-function p53 phenotypes is attributed to p53 amyloid oligomers

## Graphical Abstract

**Figure:**
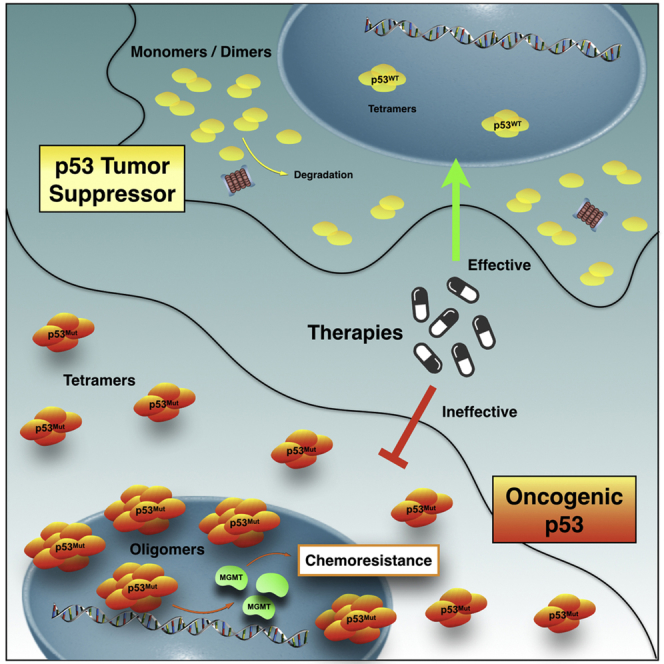

## Introduction

The p53 tumor suppressor is a key protein involved in the cellular network of different types of cellular stress (Vousden and Lane, 2007) and the most frequently mutated gene in human cancer (Bisio et al., 2014, Muller and Vousden, 2013). In response to DNA damage, oncogene activation, genotoxicity, abnormal growth signals, and hypoxia, the active tetrameric form of this protein functions as a transcription factor that regulates the expression of a large group of responsive genes involved in apoptosis, cell-cycle arrest and survival, DNA repair, genomic stability, and senescence (Vousden and Lane, 2007). TP53 mutations are associated with tumor development, and some result in p53 loss and gain of function and have a dominant-negative effect, which affects cell survival (Ano Bom et al., 2012, Ghosh et al., 2017, Lasagna-Reeves et al., 2013, Xu et al., 2011, Yang-Hartwich et al., 2015, Zhu et al., 2015).

Seminal *in vitro* biophysical studies have shown that the core domain of p53 (p53C) aggregates into a mixture of oligomers and fibrils (Ishimaru et al., 2003a). Additionally, a hotspot mutant of p53C (R248Q) was shown to seed the aggregation of the wild-type (wt) form of p53 *in vitro*, a typical behavior of a prion-like protein (Ano Bom et al., 2012, Silva et al., 2014a). Further models support the existence of coaggregation with other transcription factors (Wang and Fersht, 2015). Nevertheless, previous studies have revealed the occurrence of cell-to-cell transmissibility events involving p53 (Forget et al., 2013, Lee et al., 2009, Lee et al., 2013), which supports the suggested prion-like nature of this tumor suppressor. The ability of p53 to aggregate comes from its thermodynamic instability, which causes p53 to unfold near physiological temperatures (Bullock et al., 1997). More importantly, conformational changes in wt-p53C (the DNA-binding domain of p53) generate an aggregation-competent state, ultimately causing p53 aggregation inside the cell, which has implications for cancer establishment (Pedrote et al., 2018). For example, *in vitro* studies using the fluorescence of p53C have shown the presence of wt-p53C molten globule states prone to amyloid aggregation (Pedrote et al., 2018). In the same vein, characterization of p53 molten globule structures under mildly acidic treatment *in vitro* showed they were present in lysosomal compartments (Bom et al., 2010). NMR spectroscopy revealed molten globule-like features of p53C in association with heat shock protein 90 (Hsp90) (Park et al., 2011). Furthermore, different aggregation phenotypes were observed in biopsies of breast tumor (Levy et al., 2011) and cell lines of different cancers, including breast (Ano Bom et al., 2012), ovarian (Yang-Hartwich et al., 2015), and prostate cancers (Kluth et al., 2014), supporting the hypothesis that p53 undergoes misfolding prior to amyloid aggregation in these cells. The typical p53 pathway is controlled by the p53-MDM2 axis, triggering the proteasome-dependent degradation of p53 and surveillance by a negative feedback loop, in which p53 stimulates MDM2 transcription (Barak et al., 1993, Montes de Oca Luna et al., 1995, Wu et al., 1993). Although mutant p53 is degraded through the p53-MDM2 regulatory axis, MDM2 transcription feedback is lost, a condition that favors the escape of mutant p53 and its accumulation within the cell (Moll and Petrenko, 2003). Conceivably, the p53 structural instability and deregulation of the intracellular mutant p53 favor a condition in which conformational changes and oligomeric p53 compositions might occur, supporting oncogenic activities. Therefore, identification and analyses of the oncogenic activities in living cells related to multimeric/oligomeric mutant p53 species are urgently needed.

Glioblastoma is the most frequent, aggressive, and invasive type of brain tumor (Furnari et al., 2007, Ohgaki and Kleihues, 2007). The hallmarks of glioblastoma are uncontrolled cellular proliferation, diffuse infiltration, a propensity for necrosis, robust angiogenesis, strong resistance to apoptosis, and rampant genomic instability (Milinkovic et al., 2012). Primary and secondary glioblastoma are disease subtypes with different genetic features. A total of 90% of cases are diagnosed as primary glioblastoma without previous clinical or histological evidence (Wang et al., 2014). Approximately 30% of primary glioblastomas show TP53 mutations associated with gain-of-function, loss-of-function, and dominant-negative effects (Ham et al., 2019, Marutani et al., 1999, Wang et al., 2004, Wang et al., 2013). p53 accumulates in the cytoplasm of primary glioblastoma cells, suggesting its role in tumor pathogenesis (Nagpal et al., 2006). Notably, the M237I-p53 mutation is present in 0.63% of cancer samples (as cataloged by the International Agency for Research on Cancer, IARC). Human lymphoblast cell lines containing this mutation showed delayed X-ray-induced apoptosis (Xia and Liber, 1997) and increased chemosensitivity to temozolomide (TMZ) in glioblastoma cells after p53 knockdown (Wang et al., 2013), supporting a chemoresistance gain-of-function phenotype. Previous studies have indicated that p53 regulates the expression of the MGMT gene encoding the O^6^-methylguanine DNA-methyltransferase protein in fibroblasts and astrocytes. In glioblastoma cells bearing the M237I p53 mutation, p53 knockdown leads to a 5-fold increase in chemosensitivity to TMZ (Wang et al., 2013). The MGMT protein repairs DNA damage caused by TMZ, indicating a potential p53-dependent drug resistance mechanism. This tumor-associated mutation occurs within the Zn^2+^-binding site motif at loop 3 of p53 and drastically affects the p53 DNA-binding ability (Bullock et al., 2000). To the same extent as the hotspot mutation R175H, M237I is a destabilizing mutation that has been shown to increase solvent accessibility (Bullock et al., 2000). No mechanistic investigation has assessed the impact of increased solvent accessibility and hydration on chemoresistant p53 mutants and the consequences for protein oligomerization and p53 malignant transformation.

Here, we investigated the aggregation phenotype of a chemoresistant p53 mutant in glioblastoma cells and the ability of the mutation to promote the formation of p53 multimers that can potentially aggregate in living cells. We discovered insights into the existence of amyloid-like mutant p53 species in brain tumor cells presenting a chemoresistance gain-of-function phenotype and the distribution of mutant p53 multimers larger than the active tetrameric form of p53 in living cells. Furthermore, the destabilized mutant is found in non-native species in solution, which progressively leads to protein misfolding and amyloid aggregation. We found that increased hydration of the mutant leads to an increased tendency to aggregate. The development of personalized screening to map how tumor-associated p53 mutations affect folding, aggregation, and the malignant oncogenic phenotype of p53 is of fundamental importance toward developing therapies to avert cancer.

## Results

### Amyloid Oligomers of a Chemoresistance p53 Mutant Accumulate in Glioblastoma Brain Cell Tumors

Chemoresistance related to aberrant p53 involves the regulation of several target genes. We confirmed the increased RNA levels of MGMT and phosphatase and tensin (PTEN) (Figures S1A–S1C) and the increased cell motility (migration rate) upon scratch formation (Figures S1D and S1E) in T98G glioblastoma cells expressing a p53 protein with Met-to-Ile substitution at position 237 of p53 compared with wt-p53-expressing U87 glioblastoma cells. Furthermore, we observed increased chemosensitivity to TMZ treatment in cells expressing wt-p53 compared with cells expressing the M237I-p53 mutant (Figure S1F). Evaluating the transcriptional profile, we detected decreased p53 and PTEN RNA levels after 48 h of TMZ treatment in wt-p53-expressing cells but observed no changes in the p53 and PTEN RNA levels in glioblastoma cells carrying the M237I mutation (Figures S1G and S1H). MGMT was transcribed at higher levels during TMZ treatment in T98G cells than in wt-p53-expressing cells, in which MGMT transcription was undetectable within the studied time period (Figure S1G). These results support previous findings indicating a chemoresistance phenotype in T98G glioblastoma cells and linking the Met-to-Ile p53 substitution to this gain-of-function phenotype.

**Figure 1:**
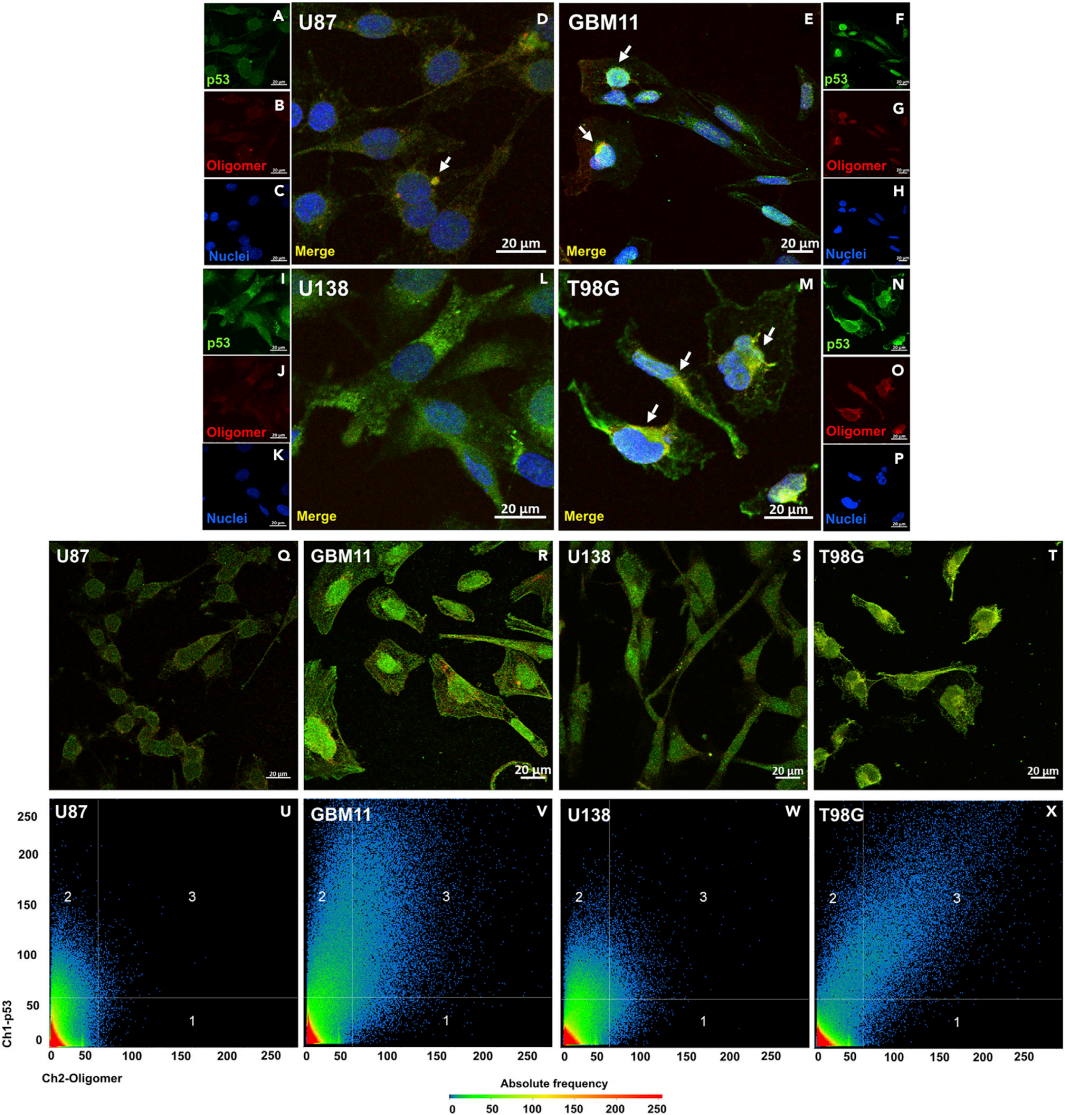
p53 Amyloid Oligomerization in Chemoresistant Glioblastoma Cells (A–P) Immunofluorescence panels for (A–D) wt p53-expressing (U87), (E–H) wt p53-expressing (GBM11), (I–L) R273H p53-expressing (U138), and (M–P) M237I p53-expressing (T98G) glioblastoma cells showing p53 colocalization (white arrows) with amyloid oligomers. Scale bars, 20 μm; (Q–X) Immunofluorescence panels for (Q) wt p53-expressing (U87), (R) wt p53-expressing (GBM11), (S) R273H p53-expressing (U138), and (T) M237I p53-expressing (T98G) glioblastoma cells showing merged p53 and amyloid oligomer channels used for pixel-by-pixel quantification. (U–X) Pixel-by-pixel correlation plots between p53 and oligomer channels. Regions 1, 2, and 3 were classified as +/−, −/+, and +/+ (p53/oligomers). Pixel frequency is color coded. Pixels within the double-positive region 3 indicate colocalization between p53 and oligomers. Three independent immunofluorescence experiments were performed with similar results. See also Figures S2 and S4.

To evaluate whether aggregation of mutant and wt-p53 has a role in the chemoresistance phenotype of glioblastoma-derived cells, we performed immunofluorescence experiments using confocal microscopy (Figures 1A–1P and S2A–S2P) of one commercial glioblastoma cell line carrying the wt-p53 sequence (U87, Figures 1A–1D), one noncommercial cell line obtained from a deceased patient with glioblastoma (GBM11, Figures 1E–1H), and two glioblastoma cell lines carrying p53 DNA-binding domain mutations; U138 cells carry the hotspot R273H mutation (Figures 1I–1L), and T98G cells exhibit TMZ chemoresistance through the M237I mutation (Figures 1M–1P). DNA sequencing of the U87, T98G, and GBM11 cells revealed that the TP53 genes of the U87 and GBM11 cells carried no mutations in the protein DNA-binding domain, in contrast to T98G cells, in which a codon change (ATG-to-ATA) at position 237 causing a Met-to-Ile substitution was confirmed (Figure S3).

We observed weak immunostaining for wt-p53 in U87 cells (Figures 1A and S2A). Controversially, for wt-p53 in GBM11 cells, diffuse immunostaining in the cytosol and stronger labeling in the nuclei were observed (Figures 1E and S2E). These wt-p53-expressing cells exhibited slight colocalization of p53 with oligomeric structures (Figures 1D and 1E), and most of the colocalized structures were located at the nuclei or in the perinuclear region (Figures 1D, 1E, S2C, S2D, S2G, and S2H). The T98G cells exhibited more colocalization of oligomeric structures and M237I-p53 in the nuclei and the perinuclear region (Figures 1M, S2O, and S2P) than U138 cells (Figures 1I–L, S2K, and S2L), revealing that the M237I-p53 mutant assumes an aggregation-prone conformation that triggers amyloid oligomerization in this chemoresistant glioblastoma cell type. Notably, accumulated M237I-p53 amyloid oligomers seem to play a specific role in the chemoresistance phenotype of these cells, as the glioblastoma cells bearing the hotspot mutant showed negligible p53 aggregation (Figures 1I–L, S2K, and S2L). The colocalization of oligomeric p53 was quantified pixel by pixel using the correlation between the fluorescent channels in different confocal images (Figures 1Q–1Y, S2Q–S2Z, and S2AA–S2FF) and Pearson's coefficient (Figures S4A–S4Q). A high number of pixels within the double-positive region (labeling number three) of the correlation plots indicates strong colocalization between p53 and amyloid oligomers in GBM11 (Figures 1R and 1V) and T98G (Figures 1T and 1Y) cells; however, the correlation between both channels (diagonal distribution of pixels) was more pronounced in glioblastoma cells expressing the M237I-p53 mutant than in those expressing wt-p53 (Figures 1T, 1Y, S2EE, and S2FF).

One typical feature of aggregated assemblies is their resistance to harsh treatments. To further support the existence of aggregated species composed of mutant p53 in T98G cells, we subjected the overall protein fraction extracted from T98G cells to p53 immunoprecipitation followed by SDS-denaturing gels and immunoblots to detect p53. We observed SDS-resistant, high-molecular-weight mutant p53 species using this approach (Figure S5A). In addition, we observed a diffuse band at approximately 37 kD, which likely represented proteolytic M237I p53 fragments. In addition, whole H1299 and T98G cell extracts were subjected to size exclusion chromatography (Figure S5B), and eluted fractions were assessed by dot blots to detect p53 species (Figure S5C). Eluted fractions from T98G cells corresponding to the column void volume showed positive immunostaining for p53 species (Figure S5C), confirming the presence of high-molecular-weight p53 assemblies in this glioblastoma cell type.

### Excess Formation of Mutant p53 Species Sustains Aggregation in Glioblastoma Cells

**Figure 2:**
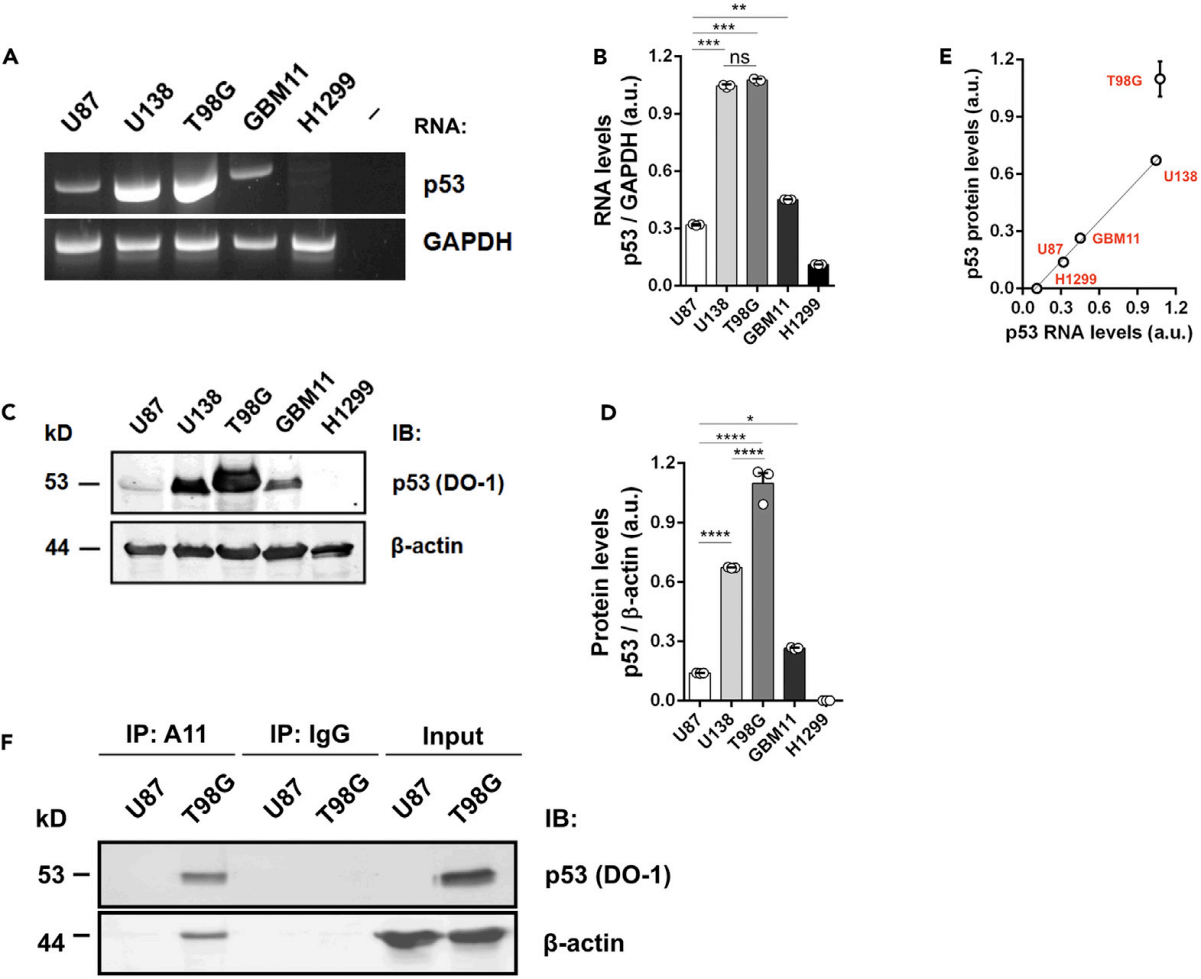
Accumulation of M237I-p53 in Glioblastoma Cells (A) Agarose strips showing the p53 RNA levels in glioblastoma and H1299 cells. (B) Dot plots showing densitometry values obtained after normalizing p53 transcriptional levels to those of glyceraldehyde 3-phosphate dehydrogenase, GAPDH. The results are shown as the mean ± SEM of three densitometry measurements. Experiments were performed twice with similar results (***U87 versus U138, p = 0.0005; ***U87 versus T98G, p= 0.0002; **p= 0.0012, and ns not significant). (C) Immunoblots (IBs) showing total p53 levels (DO-1) and β-actin as a loading control. (D) Dot plots showing densitometry values obtained after normalizing p53 protein levels to those of actin. The results are shown as the mean ± SEM of three densitometry measurements. Experiments were performed twice with similar results (****p< 0.0001, *p= 0.0365). (E) Correlation plot of p53 RNA versus p53 protein levels in glioblastoma and H1299 cells. (F) Immunoblots (IBs) showing total p53 levels (input) and the levels of oligomeric p53 in T98G cells after immunoprecipitation (IP) with an oligomer-sensitive antibody (A11). See also Figure S6. kD refers to the expected weight of the detected proteins in kilodaltons.

As protein accumulation favors misfolding and ultimately aggregation and cell deregulation, we evaluated the p53 RNA and protein levels in glioblastoma cells expressing wt (U87 and GBM11) and mutant (U138 and T98G) p53 (Figures 2A–2D and S6). Surprisingly, we observed increased p53 expression at both the RNA and protein levels in the chemoresistant M237I-p53-expressing cells (Figures 2A–2D). As shown in Figure 2A, the p53 RNA levels in mutant p53-expressing cells (U138 and T98G) were higher than those in wt-p53-expressing cells (U87 and GBM11). Densitometric values (Figures 2B and 2D) for the p53 RNA and protein levels were expressed in correlation plots in which the p53 protein/RNA levels in T98G cells were different from those in the other cells, suggesting p53 deregulation at the protein level (Figure 2E). The levels of M237I-p53 in T98G cells were 27.2% higher than M237I-p53 RNA levels, suggesting p53 accumulation in these cells. To further confirm M237I-p53 oligomeric species in this chemoresistant cell line, we performed immunoprecipitation of whole U87 and T98G cell extracts using an oligomer-sensitive antibody followed by p53 immunoblot detection. The results revealed stronger p53 immunostaining in T98G than U87 cells (Figures 2F and S6) consistent with the immunofluorescence results (Figures 1D and 1M).

To investigate whether the increased M237I-p53 accumulation is potentially related to the presence of unfolded p53 species and ER stress, we detected the protein levels of two unfolded protein response (UPR) sensors, inositol-requiring protein-1a (IRE-1α), and protein kinase RNA-like ER kinase (PERK). In contrast to U138 and GBM11 cells, T98G cells expressed detectable levels of the IRE-1α and PERK sensors, as shown by immunoblots (Figures S7A–S7D). Notably, U87 cells showed significantly higher levels of these UPR sensors than other cells, but as the p53 level was negligible, ER stress cannot be attributed to wt-p53 overload in this cell line. Furthermore, we detected equally higher Hsp90 levels in both the M237I-p53-expressing cells and U87 cells expressing wt-p53 than the Hsp90 levels in the other studied cells (Figure S7D). Altogether, the accumulation of M237I-p53 (Figure 2) species in glioblastoma cells and the presence of amyloid p53 oligomers (Figures 1, S2, and S4) potentially link oligomeric p53 mutant compositions to chemoresistance gain-of-function activity in glioblastoma.

### The Mutant p53 Oligomer Composition in Living Cells

**Figure 3:**
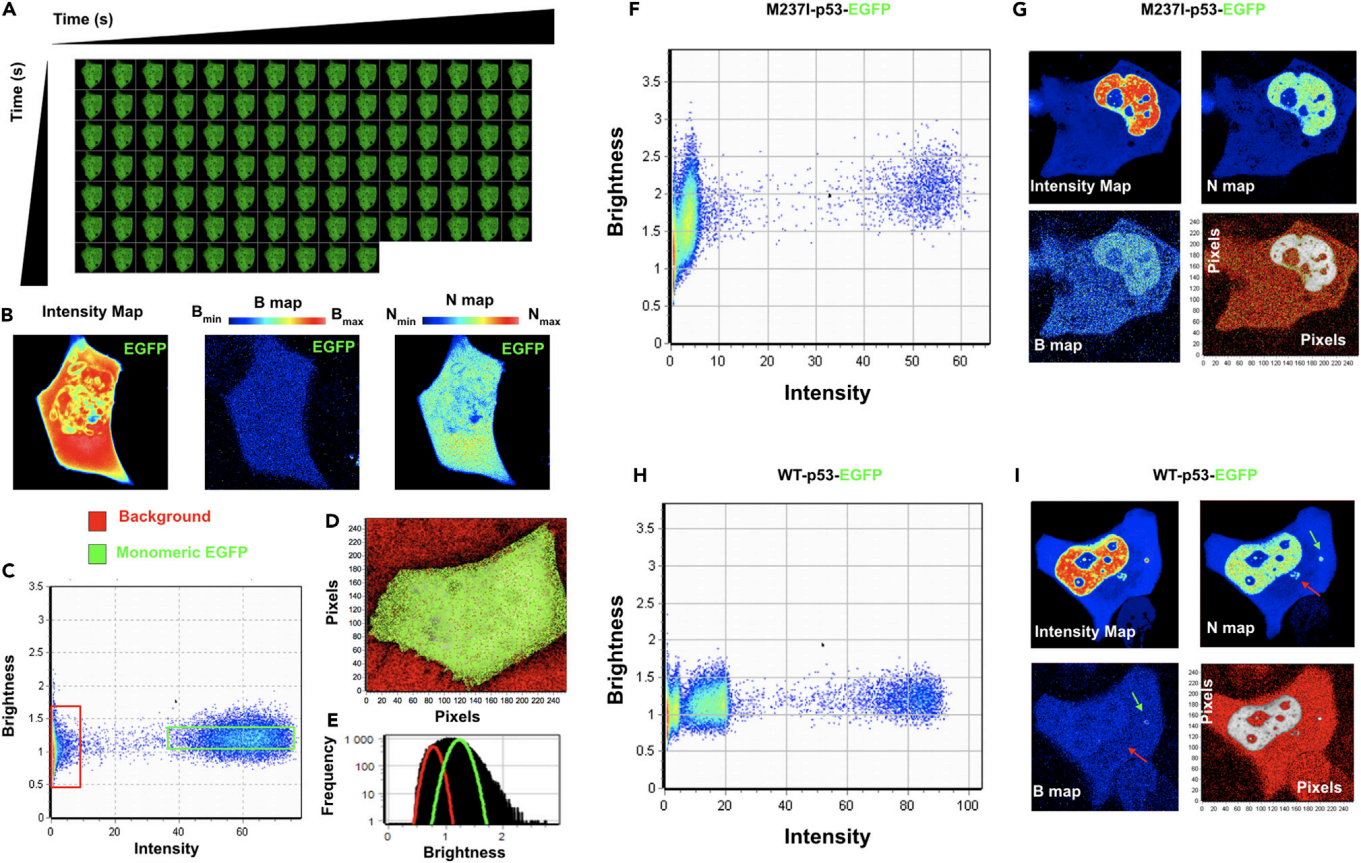
Detection of p53 Oligomers in Living Cells (A) Representative frames of an EGFP-transfected H1299 imaged cell used as input for statistical number and brightness (N&B) analysis. (B) Representative intensity, brightness (B), and number of molecule (N) maps of an EGFP-transfected H1299 imaged cell. (C–E)(C) Selection of pixels within brightness plots (red and green squares) (D) or from Gaussians (red and green) of brightness distribution, which can be used to (E) depict their distribution in imaged cells. Red colors correspond to the immobile fraction (background), and green pixels correspond to monomeric EGFP. (F–I) (F and G) and (H and I), Brightness plots of M237I-p53 and wt-p53, respectively, showing different brightness distributions. Intensity, B, and N maps of imaged (F and G), M237I-p53- and (H and I) wt-p53-expressing cells highlighting bright pixels indicating M237I-p53 within the nuclei (white arrows). Puncta of wt-p53, in which an increased number of molecules in N maps do (green arrows) or do not (red arrows) overlap with bright pixels are shown. Red pixels in the bottom right panels of imaged cells represent cytosolic (red) and nuclear (green) p53 distribution according to Gaussian brightness histograms. All N&B experiments were performed at least twice with similar results. See also Figures S9–S11.

Because M237I-p53 forms amyloid oligomers in glioblastoma cells expressing a chemoresistance gain-of-function phenotype, we decided to explore the oligomer composition and distribution of this mutant in living cells. We first detected the different distributions of the EGFP-tagged wt-p53 and M237I-p53 constructs in H1299 cells (Figure S8). Next, using systematic number and brightness (N&B) analysis of fluorescence fluctuation spectroscopy data, we directly measured the wt- and M237I-p53 oligomerization status and distribution within living cells. To exclude any interference from endogenous p53 aggregation, we used p53-null H1299 lung carcinoma cells. N&B statistical analysis provides a pixel-by-pixel resolution map of the molecular oligomers in living cells by measuring the average intensity and variance in each pixel in a time series of images (Digman et al., 2008, Digman et al., 2013) (Figures 3A, S9A, and S9B). From these values, the apparent brightness (B maps) and average number of molecules (N maps) can be estimated (see Transparent Methods). During the acquisition of image frames, the laser power was set accordingly to eliminate a contribution from photobleaching (see Transparent Methods and Figures S9C and S9D). The brightness maps directly report the oligomerization status of proteins, as larger particles generate higher fluorescence fluctuation as they pass in and out of the excitation volume, resulting in higher variance. Thus, the higher the apparent brightness is, the higher the oligomerization status is, and the fewer molecules there are within the excitation volume.

To compare wt and mutant p53 oligomeric states within living cells, we first normalized the brightness levels of the monomeric EGFP standard (Figures 3A–3E). We obtained an average B value for monomeric EGFP of 1.21 (n = 7 imaged cells, Figures 3C–3E and S10). Cells transfected with EGFP showed a homogeneous distribution of monomeric EGFP throughout the whole cell (Figures 3D, S10, and S11A). Based on the normalized B value of monomeric EGFP, the oligomeric status and distribution of M237I-p53-EGFP-transfected (Figures 3F, 3G, and S12A) and wt-p53-EGFP-transfected (Figures 3H, 3I, and S12B) cells were evaluated. Brightness plots of M237I-p53-transfected (Figures 3F and 3G) and wt-p53-transfected (Figures 3H and 3I) cells revealed distinctly different distributions (n = 10 imaged cells, Figures 3, S11B, S11C, and S12). M237I-p53-transfected cells exhibited pixels with higher B values than those of wt-p53-transfected cells (Figures 3F–3I and S12). B maps of the imaged cells showed that 9 of 10 M237I-p53-transfected cells had brighter pixels than wt-p53-transfected cells either within the nuclei or throughout the entire cytosol (Figures 3F–3I and S11C, white arrows). In contrast, wt-p53-transfected cells showed a more homogeneous B map distribution than M237I-p53-transfected cells (Figures S11B and S11C), which is similar to the findings in monomeric EGFP-transfected cells (Figure S11A). Among 10 imaged wt-p53-transfected cells, one exhibited brighter pixels within the nuclear region than the imaged M237I-p53-transfected cells (Figure S11B, red asterisk). Interestingly, the distribution of abundant wt-p53 molecules confined within cytosolic puncta does not predominantly overlap with the bright pixel regions (Figures 3F–3I, S11B, and S12, red arrows), although this finding was not true in all cases (Figures 3F–3I and S11B, green arrows). Most of the wt-p53 molecules within the puncta overlapped with bright pixels corresponding to assemblies of higher-order aggregates with B values in the range of 3–4 (Figure S13). In M237I-p53-expressing cells, puncta formation was not observed in the imaged cells and the increased number of M237I-p53 molecules within the nuclei predominantly overlapped with bright pixels.

**Figure 4:**
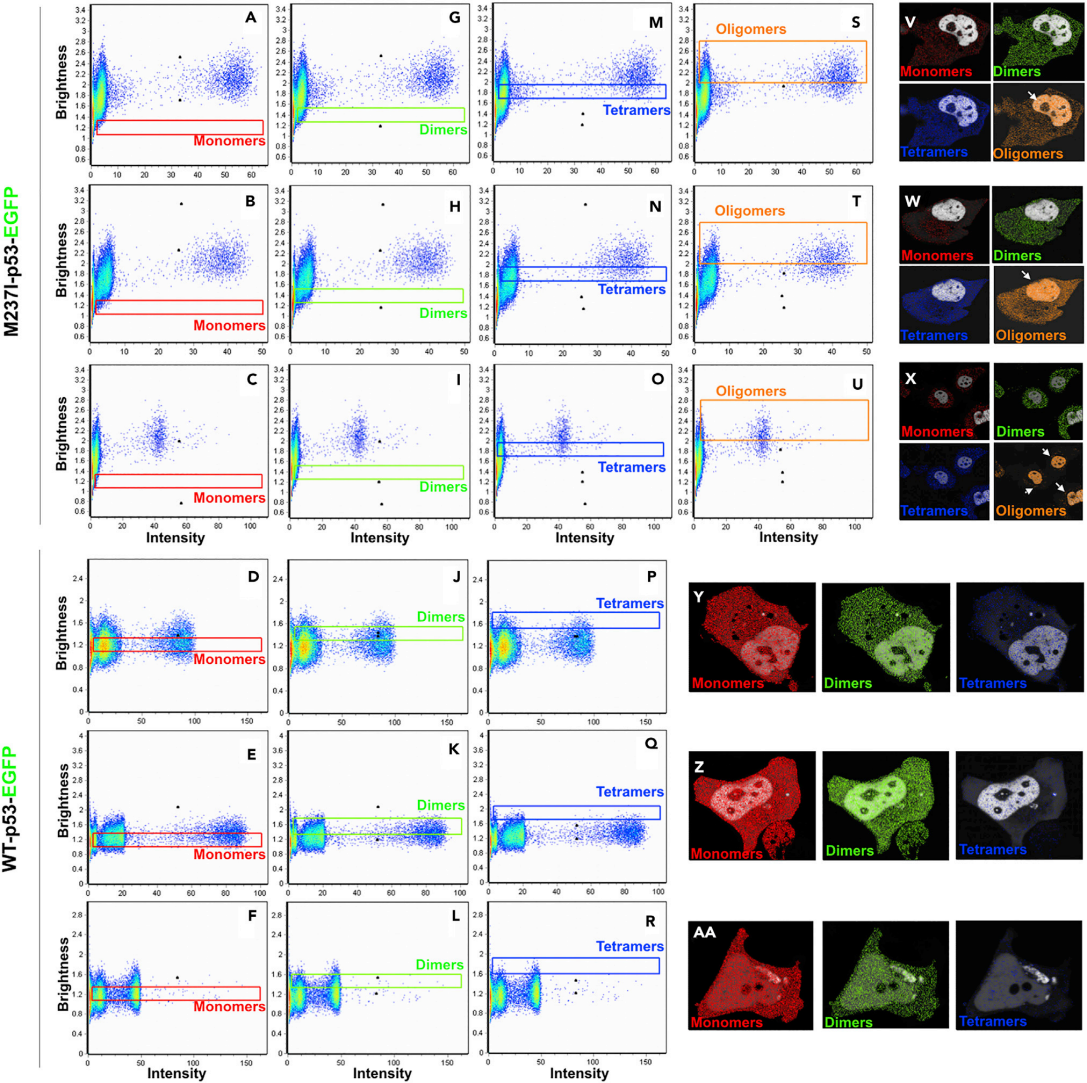
The Composition of Mutant p53 Oligomers in Living Cells (A–U) Selection of pixels showing the brightness levels of M237I- and wt-p53 (A–F) monomers (red), (G–L) dimers (green), (M–R) tetramers (blue), and (S–U) M237I-p53 oligomers (orange). (V–AA) Distribution of selected pixels in imaged cells expressing (V–W) M237I-p53 and (Y–AA) wt-p53. White arrowheads highlight the predominant distribution of M237I-p53 oligomers in cellular nuclei. The same color code is used to report oligomeric states throughout the figure. All N&B experiments were performed at least twice with similar results. See also Figures S12 and S13.

To precisely assess the distinct oligomer composition and distribution of wt- and M237I-p53 in living cells, we used the normalized apparent brightness of monomeric EGFP (B value standard = 1.2) and the following equation: B = εn + 1 (see Transparent Methods) and estimated B values of 1.4 and 1.8 for p53 dimers and tetramers, respectively. This implies that B values higher than 2 correspond to species larger than p53 tetramers. We used rectangular slices in brightness plots to indicate the brightness of monomeric (Figures 4A–4F, red), dimeric (Figures 4G–4L, green), and tetrameric (Figures 4M–4R, blue) wt- and M237I-p53 molecules and the brightness levels for M237I-p53 oligomers (Figures 4S–4U, orange). Pinpointing selected pixels with different B levels in the imaged cells allowed us to make important conclusions about the distribution and behavior of wt- and M237I-p53 oligomers. M237I-p53 showed very low cytosolic B levels corresponding to monomers and dimers, in contrast to the pronounced cytosolic distribution of M237I-p53 tetramers (Figures 4V–4W). Surprisingly, most B values corresponding to oligomeric M237I-p53 molecules (at least larger than tetramers) were widely distributed within the nuclei and less densely distributed throughout the cytosolic region (Figures 4V–4W, white arrowheads). Meanwhile, wt-p53 B values corresponding to monomers and dimers were predominant, and wt-p53 was mostly distributed throughout the entire cell, whereas tetramers were extremely sparse (Figures 4Y–4AA). Altogether, these results provide direct evidence of mutant p53 oligomers (larger than tetrameric p53) within the nuclei of living cells. More important, the mutant p53 used in these studies forms amyloid oligomers in glioblastoma cells that present a chemoresistance gain-of-function phenotype, a condition conducive to the formation of misfolded species.

### Increased Hydration Explains the Reduced Structural Integrity and Amyloid Oligomerization of Mutant p53

**Figure 5:**
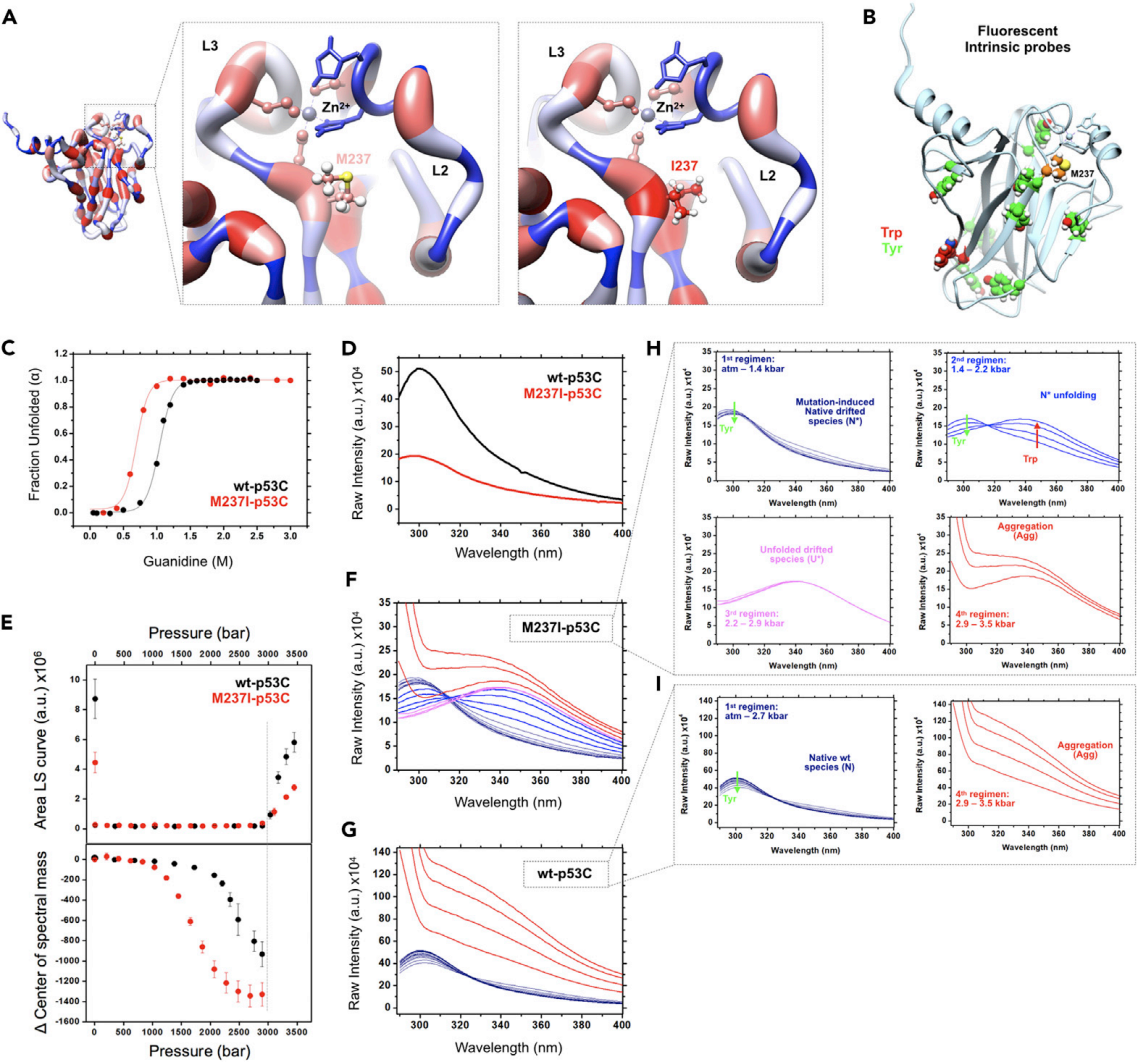
Hydrated Mutant p53 States Lead to Aggregation (A) Hydrophobic maps highlighting increased hydrophobicity within the Zn^2+^-binding structural motif after Ile237 introduction. (PDB: 2FEJ) (B) Schematics showing intrinsic tyrosine (green spheres) and single tryptophan (red spheres) fluorescent probes used to report conformational changes in the p53 DNA-binding domain. Methionine 237 is highlighted in orange. (PDB:2FEJ) (C) Plot of the fraction of unfolded M237I-p53C and wt-p53C against increasing concentrations of guanidine hydrochloride (Gu-HCl). (D) Tyrosine fluorescence emission spectra of M237I- and wt-p53C. (E) Pressure jump experiments reporting light scattering (LS, top) and changes in the center of spectral mass of M237I- and wt-p53C. Increased LS measurements at atmospheric pressure represent values obtained after depressurizing the samples. (F and G) Collection of fluorescence emission spectra for (F) M237I-p53C and (G) wt-p53C, respectively. (H and I) Highlights in the different spectral regimens observed for (H) M237I-p53C and (I) wt-p53C, respectively. See also Figures S14A and S14B. The results are shown as the mean ± SEM of at least three independent experiments.

To identify the structural determinants causing the distinct composition of mutant p53 oligomers in cells, we performed *in vitro* and structural analyses. Met237 is located at the beginning of the L3 loop close to the Zn^2+^-binding site, which is similar to the location of the hotspot mutation R175H (Figure S14A). The S-methyl thioether side chain of Met237 is in close proximity to the His179 imidazole group participating in the Zn^2+^ coordinate sphere (Figure S14A). Evaluation of wt- and M237I-p53 hydrophobicity maps revealed that the small increase in hydrophobicity within the mutant p53 Zn^2+^-binding site explains its destabilization (Figure 5A). *In silico* mutagenesis showed that, for correct accommodation, the bulky isoleucine side chain must move away from the Zn^2+^-binding site to avoid clashes (Figures 5A and S14A). This situation potentially impacts the Met237-Cys238-Zn^2+^ hydrophobic environment and proper zinc coordination within the L2-L3 interface loops. Furthermore, a stabilizing H-bond occurring between Met237 and Leu194, which is located at the base of loop L2, is disrupted by amino acid substitution. Because Zn^2+^ has a structural role in p53 and the hydrophobic environment of Met237 seems to promote proper Zn^2+^ accommodation, we performed guanidine hydrochloride titrations and detected the fluorescence of intrinsic p53 probes to determine the structural stability of recombinantly expressed M237I-p53C compared with that of wt-p53C (Figures 5B and 5C). The concentration of guanidine at the midpoint of the folding transition (G_50%_) was approximately 0.7 and 1 M for M237I-p53C and wt-p53C, respectively, confirming the structural role of M237 in maintaining the p53 structural stability (Figures 5C and S14B). This mutation affects the conformational ensemble of M237I-p53C native states (N), as the M237I-p53C Tyr fluorescence emission spectra was 60% less intense than that measured for wt-p53C (Figure 5D). As the solvent polarity was equal in these measurements, the most likely explanation for the pronounced reduction in intensity is the dipolar reorientation of p53 Tyr probes upon excitation due to increased protein hydration.

To test the effects of hydration on the p53C domain and how hydration impacts the p53 Cys_3_-His_1_ Zn^2+^-binding motif, we designed pressure jump experiments reporting changes in the fluorescence of p53 intrinsic probes. Interestingly, M237I-p53C was more sensitive to pressure-induced treatment than wt-p53C (Figure 5E, bottom). The p_50%_ of M237I- and wt-p53C was approximately 1.6 and 2.6 kbar, respectively, confirming that the hydrophobic Met-to-Ile substitution within the Zn^2+^-binding motif triggers the formation of an ensemble of destabilized and hydrated structural conformers. The reduced intensity of the Tyr residues at atmospheric pressure in M237I-p53C indicates the movement of these residues to a more hydrated environment with no predominant loss of tertiary structure, as no redshift was observed within the emission spectra (Figure 5D). The hydrophobic introduction of Ile237 within the Zn^2+^-binding structural motif was sufficient to create an ensemble of destabilized and hydrated conformers prone to amyloid aggregation. If true, it is reasonable to claim that M237I-p53C contains exposed hydrophobic patches. More interesting is the identification of different emission spectral regimens for M237I-p53C (Figure 5F) and wt-p53C (Figure 5G). We are aware that the ensemble of M237I-p53C structural conformers (hereafter called N∗) is not the same as the wt-p53C native ensemble (hereafter called N) but is instead composed of a broad population of destabilized native-drifted states exhibiting increased hydration. In contrast to the spectral regimen observed for wt-p53C, we identified four spectral regimens for M237I-p53C. The first regimen was common to M237I-p53C (up to 1.4 kbar) and wt-p53C (up to 2.7 kbar), in that there was no appreciable difference in the Tyr emission spectra between the M237I-p53C N∗ and wt-p53C N states (Figures 5H and 5I; first regimen). The destabilized and hydrated M237I-p53C N∗ states behaved differently from the wt-p53C N states within a range between 1.4 and 2.2 kbar. The N∗ states exposed a single Trp residue, a clear indication of protein unfolding (Figure 5H; second regimen). Based upon the exposure of the p53 intrinsic probes, pressure-induced unfolding of wt-p53C N states did not occur, and spectral signals moved directly to the fourth regimen, in which protein aggregation occurred (Figure 5I; fourth regimen). Aggregation was shown by the increased scattering contribution within the 300- to 310-nm range (Figure 5I; fourth regimen) and confirmed by light scattering (LS) measurements (Figure 5E, top). Full unfolding of the M237I-p53 N∗ states was achieved between 2.2 and 2.9 kbar when the spectral signals reached a plateau (Figure 5H; third regimen). This pressure range supports a population of unfolded-drifted species (U∗), as small increments in pressure led to aggregation (Figure 5H; fourth regimen and Figure 5E, top).

**Figure 6:**
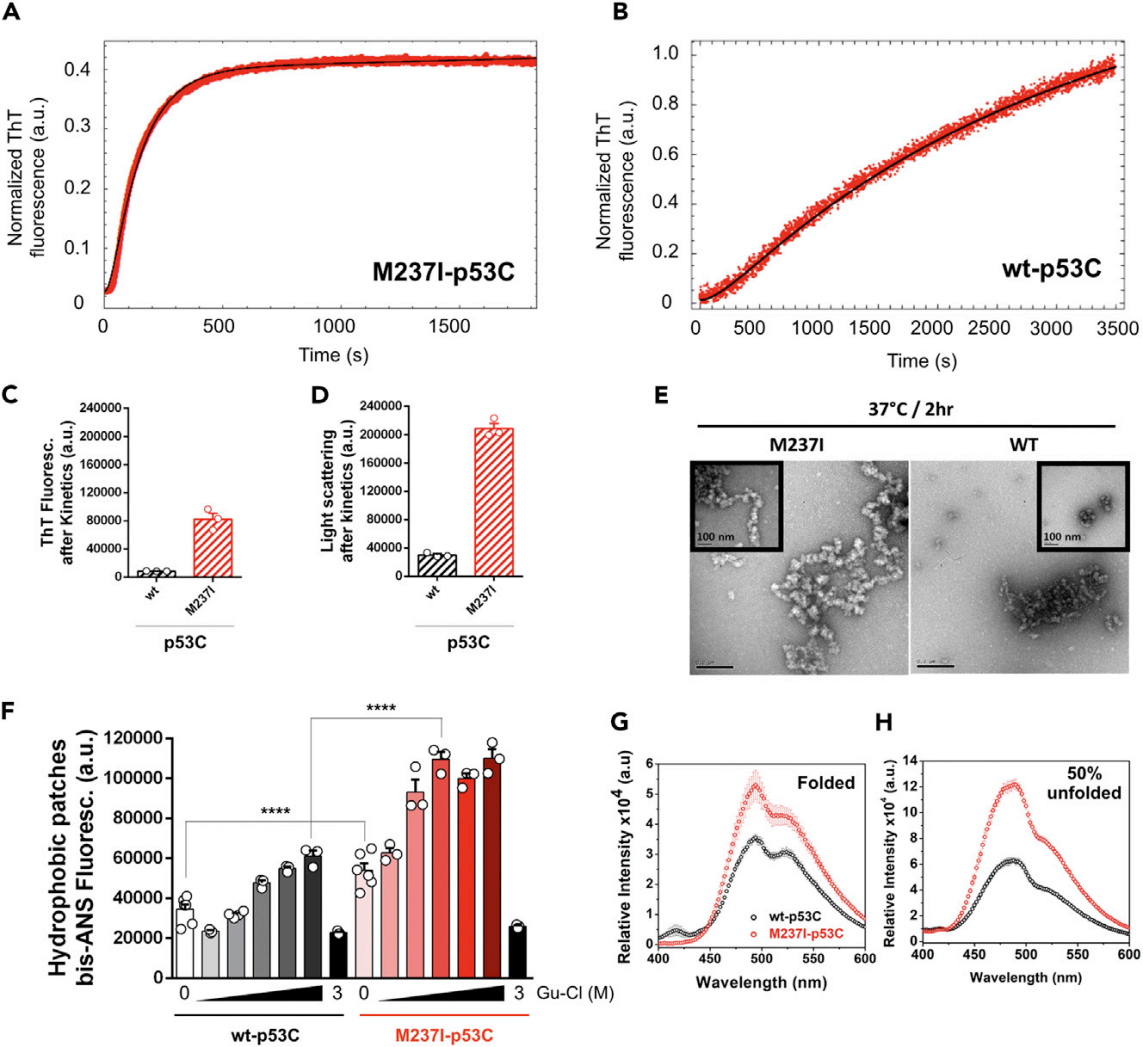
Amyloid Aggregation of M237I-p53 in Solution (A and B) Normalized thioflavin T (ThT) fluorescence kinetics of (A) M237I- and (B) wt-p53C at physiological temperature. The k1 and k2 rate constants (Table 1) were obtained after fitting (black lines) the experimental points (red) using first-order lag kinetics. (C and D) Dot plots showing (C) ThT fluorescence and (D) light scattering (LS) values after kinetic traces for wt- and M237I-p53C, respectively. (E) Negative staining micrographs after kinetic traces for M237I- and wt-p53C. Insets show zoomed areas. Scale bars, 0.2 μm. (F) Dot plot reporting the fluorescence of bis-ANS and wt- and M237I-p53C hydrophobicity following exposure to the solvent under increasing concentrations of guanidine hydrochloride, Gu-HCl (0, 0.2, 0.4, 0.6, 0.8, 1, and 3 M). The results are shown as the mean ± SEM of at least three independent experiments (p< 0.0001, folded wt-p53 versus folded M237I-p53 and wt-p53 G_50%_ versus M237I-p53 G_50%_). (G and H) bis-ANS fluorescence spectra of wt- and M237I-p53C proteins under (G) folded and (H) 50% unfolded (at the G_50%_) conditions, respectively. See also Figures S14C and S14D.

To demonstrate that the conformational changes observed in the destabilized non-native species result in amyloid aggregation, we measured the kinetics of M237I-p53C by scattering and thioflavin T (ThT). In contrast to what was observed for wt-p53C, M237I-p53C revealed a very short lag phase (∼20 s) followed by a sharp increase in both signals up to a plateau at ∼500 s (Figures 6A and 6B, S14C, and S14D). In the case of wt-p53C, the lag phase was longer (∼250 s) than that of M237I-p53C and followed by a smooth increase in scatter and the ThT signal. Fitting the curves to first-order lag kinetics revealed higher rate constants for the M237I-p53C than wt-p53C (Table 1), suggesting the fast formation of a population of aggregation-competent M237I-p53C conformers at physiological temperature. The ThT fluorescence levels of M237I-p53C were significantly higher than those of wt-p53C, revealing increased levels of M237I-p53C amyloid-like species even though a mixture of amyloid-like forms and general aggregation (amorphous + amyloid) was present, as evidenced by LS (Figures 6C and 6D). Negative staining transmission electron microscopy showed higher-order assemblies similar to amyloid-like structures composed of the M237I-p53C (Figure 6E). Finally, by treating wt and M237I-p53C with mild concentrations of guanidine, we observed increased exposure of hydrophobic patches in M237I-p53C compared with that in wt-p53C (Figure 6F). Bis-ANS emission spectra showed increased M237I-p53C hydrophobic exposure both in the folded state and at approximately 50% unfolding (Figures 6G and 6H). These findings confirmed that, by perturbing overall p53 hydration and protein hydrophobicity to the solvent, the M237I substitution populates destabilized native-drifted species that are preamyloidogenic. Collectively, these findings support a model in which destabilized aggregation-prone species are formed and contribute to the distinct composition of an agglomeration of chemoresistance mutant p53.

**Table 1:** Kinetic Parameters according to First-Order Lag Kinetics

	WT-p53C	M237I-p53C
k_1_ (min^−1^)	4.1×10^−4^± 3.7 × 10^−6^	0.481 ± 0.007
k_2_ (min^−1^)	5.9×10^−3^±2.7 × 10^−4^	2.371 ± 0.097

## Discussion

The aggregation phenotypes of p53 observed in breast, ovarian, and prostate tumors have been correlated with p53 dominant-negative and oncogenic gain-of-function activities (Kluth et al., 2014, Lasagna-Reeves et al., 2013, Xu et al., 2011). However, there has been no information that p53 can form oligomeric species in glioblastoma tumors and that agglomeration is associated with chemoresistance oncogenic activity in this type of brain tumor. Here, we explored questions related to the chemoresistance phenotype involving p53 in glioblastoma with the following aims: (1) identify p53 amyloid oligomers in glioblastoma cells, (2) investigate the composition and distribution of chemoresistant mutant p53 oligomers in living cells, (3) determine whether misfolding is a prerequisite for aggregation in cells, and (4) determine the mechanism by which mutant p53 amyloid aggregation occurs in solution.

The chemoresistance phenotype to TMZ, the standard treatment for glioblastoma, has been linked to a p53-specific mutation in glioblastoma cells through the increased expression of O^6^-methylguanine DNA-methyltransferase (MGMT), an enzyme that repairs DNA damage caused by TMZ (Wang et al., 2013). Accordingly, the homologous PTEN tumor suppressor was found to exert oncogenic effects in mutant p53-expressing glioblastoma cells, including cell cycle progression, cell proliferation, and cell death inhibition (Li et al., 2008). Compared with those in wt p53-expressing glioblastoma cells, significantly higher transcript levels of MGMT and PTEN and faster migration in glioblastoma cells with the M237I-p53 mutation that are insensitive to TMZ were detected. The increased MGMT and PTEN transcript levels in T98G cells support their oncogenic transformation, which is reflected by the acquisition of phenotypes such as chemoresistance and acceleration of cell proliferation and migration.

Several lines of evidence have indicated that amyloid-like p53 oligomers are present in different tumor cells and tissues, as shown by using an amyloid oligomer-sensitive antibody (Ano Bom et al., 2012, Ghosh et al., 2017, Levy et al., 2011). The TMZ-chemoresistant glioblastoma cells showed higher levels of mutant p53 amyloid oligomers than glioblastoma cells expressing either wt p53 or a hotspot p53 mutant not associated with chemoresistance. Abnormal accumulation of wt and mutant p53 was described in different types of cancer cells, such as neuroblastoma, retinoblastoma, breast cancer, and colon cancer cells (Kluth et al., 2014, Levy et al., 2011). However, the levels of accumulated wt p53 and p53 mutants drastically differ, with significantly increased accumulation of the latter proteins. Aggregation of mutant p53 has been shown to impair the tumor suppressor activities of p53 and contribute to the gain of oncogenic phenotypes including chemoresistance. In high-grade serous ovarian carcinoma, p53 aggregation is related to platinum resistance (Yang-Hartwich et al., 2015). Our findings link TMZ chemoresistance in glioblastoma to a mutant-specific p53 aggregation phenotype.

One of initial modifications required for dissociation of the p53 negative regulator MDM2 and activation of transcriptional activity is p53 phosphorylation at Ser15 (Dumaz and Meek, 1999, Shieh et al., 1997). Furthermore, cellular homeostasis is dictated by protein synthesis, degradation, and repair. When protein folding efficiency is threatened owing to protein overload within the ER, cells experience ER stress and activate the UPR (Hetz, 2012, Walter and Ron, 2011). If mutant p53 production exceeds the rates at which ER quality checks manage recently synthesized proteins, cells face a bottleneck in protein control. The expression of the two UPR sensors, inositol-requiring protein-1a (IRE-1a) and protein kinase RNA-like ER kinase (PERK), supports ER stress due to the overload of misfolded mutant p53 species. These two sensors participate in prosurvival signaling to eliminate misfolded species, reduce the transcription of mRNAs and increase the concentration of molecular chaperones to process accumulated proteins within the ER (Hetz et al., 2011).

Hsp90 is an ER-resident molecular chaperone that promotes proper folding and maturation of client proteins (Zhao et al., 2005). However, Hsp90 was previously shown to participate in the adaptive response of cancer cells by stabilizing and preventing degradation of overexpressed mutant p53 (Finlay et al., 1988, Whitesell et al., 1997, Whitesell et al., 1998). We speculate that Hsp90 might fail to chaperone the chemoresistance mutant p53, but the expression of equally high Hsp90 levels in U87 (wt-p53) and T98G cells indicate that other escape mechanisms may favor M237I accumulation and aggregation. Unfortunately, we were not able to rule out other potential escape mechanisms, such as impairment of the p53 degradation system. Hsp90 has been shown to chaperone recombinant wt p53 by positively modulating its DNA-binding ability at physiological temperatures (Walerych et al., 2004), and the DNA-binding domain of p53 adopts a molten globule-like state as it transiently binds to Hsp90 (Park et al., 2011). Recently, long-term Hsp90 inhibition was shown to extend the survival of R248Q p53-and R172H p53-expressing mice but not their corresponding p53(−/−) littermates (Alexandrova et al., 2015). Hsp90 and other cochaperones can also effectively distinguish wt p53 and mutant p53 conformations, as Hsp90 selectively binds to wt p53 but not to the hotspot mutant R175H p53, and other cochaperones are first required prior to the Hsp90-R175H p53 interaction (King et al., 2001). The effects of p53 mutations on its structure may trigger a divergent chaperone cellular response impairing proper p53 turnover. In support of this finding, the Hsp90 inhibitor geldanamycin was previously shown to either stimulate cytoplasmic mutant p53 translocation into the nucleus or increase mutant p53 ubiquitination and proteasome-mediated degradation (Whitesell et al., 1997, Whitesell et al., 1998). Notably, some destabilized mutant p53 proteins exhibit conformational changes that are sufficient to threaten chaperone activity and enhance aberrant stability.

The differences in the oligomer composition and localization of the wt and M237I p53 oligomers in living cells are also intriguing. Notably, the oligomeric species detected by N&B analysis in living cells are not necessarily the same as those amyloid oligomers revealed by immunofluorescence assays. Amyloid species detected by the oligomer-sensitive antibody are likely large species owing to their close association with chaperones or other p53 family members (Xu et al., 2011). Meanwhile, those observed by N&B most likely represent the initial association steps of p53 multimers, such as octamers or limited multimers. In support of this finding, p53 octamers were recently shown to bind p53 response elements (REs) containing long spacers (Vyas et al., 2017). It is tempting to speculate that destabilized p53 mutations such as M237I would favor p53 octamers to form rapidly and bind to REs related to gain-of-function phenotypes. However, the identification of amyloid features of these initial multimers requires further investigation. We present evidence of oligomers consisting of a chemoresistant mutant p53 that are larger than the active p53 tetrameric form inside the nuclei of living cells (Figures 3 and 4). Although a previous report using N&B analysis showed that the balance among wt p53 monomers, dimers, and tetramers is not determined simply by p53 concentration (Gaglia et al., 2013), our results indicate that oligomers formed by cancer-associated mutations are concentration-dependent. In contrast to wt p53, for which we mostly detected monomers and dimers throughout the cytosol and nucleus, M237I p53 tetramers were abundant in the cytosol, and M237I p53 oligomers (at least those larger than tetramers) were densely distributed within the nucleus (Figure 4). As DNA damage was not chemically induced in our cells, we considered the imaged cells to be under resting conditions. Consistent with this finding, the distribution of wt p53 monomers and dimers conformed to previous estimations (Gaglia et al., 2013). Surprisingly, M237I p53 tetramers were present in the cytosol. The tetramerization domain of p53, which contains a nuclear export signal (NES, residues 340–351), has been shown to be occluded as tetrameric p53 is formed, which forces p53 tetramers to remain in the nucleus (Stommel et al., 1999). According to this model, the monomeric and dimeric forms of p53 would have an exposed NES motif, allowing them to be shuttled to the cytosol. Based on our observations of M237I p53 tetramers in the cytosol, we speculated that M237I p53 dimers present in the cytosol adopt a conformation amenable to the formation of stable tetramers. Given the potential non-transcriptional roles of p53 (Green and Kroemer, 2009), the presence of functional mutant p53 tetramers in the cytosol of cancer cells may represent an unsolved conundrum that should be investigated. Furthermore, because the oligomerization status of p53 is linked to the cell fate, as p53 dimers show growth arrest but not apoptotic functions like tetramers (Fischer et al., 2016), the presence of p53 tetramers in the cytosol and larger oligomers in the nuclei of cancer cells may help us to understand the oncogenic activities in cancer. A recent contribution from the Prives group showed that the oligomerization status of p53 substantially impacts p53-MDM2 regulation and p53 localization, raising questions about how p53 tetramers are degraded (Katz et al., 2018). The functionality of oligomers larger than tetramers in the nucleus is challenging to address, but based on previous works from our group (Ano Bom et al., 2012, Bom et al., 2010, Ferraz da Costa et al., 2018, Ishimaru et al., 2009, Ishimaru et al., 2003a, Ishimaru et al., 2003b, Levy et al., 2011, Pedrote et al., 2018, Rangel et al., 2019, Silva et al., 2018) and others (Ghosh et al., 2017, Kluth et al., 2014, Lasagna-Reeves et al., 2013, Soragni et al., 2016, Xu et al., 2011, Yang-Hartwich et al., 2015), we believe these oligomers participate in oncogenic transformation and cancer gain-of-function phenotypes. The oligomers identified in this work are formed by a chemoresistant mutant p53 present in glioblastoma tumors. It is tempting to suggest that the cellular environment limits misfolding and massive aggregation, especially by interacting with chaperones. This would lead to the formation of small oligomers that might exert gain-of-function effects. Notably, the nucleus is full of nucleic acids and especially RNAs, which were recently shown to limit p53 aggregation but facilitate small oligomer formation (Kovachev et al., 2017).

The presence of M237I p53 amyloid oligomers in glioblastoma cells (Figure 1) and the nuclear M237I p53 oligomers captured in live cells (Figures 3 and 4) indicate that mutant p53 destabilized conformations are present in cells and that, by evading the protein control apparatus, these aberrant p53 forms undergo oligomerization. Therefore, it is important to explore the mechanisms by which cancer-associated p53 mutations affect its folding. The polypeptide fold is extremely dependent on either its amino acid composition or the environment surroundings. The hydration of nonpolar amino acids occurs prior to amyloid aggregation and is often assessed by pressure perturbation experiments (Cordeiro et al., 2004, de Oliveira and Silva, 2015, Ferrão-Gonzales et al., 2003, Silva et al., 2014b, Silva et al., 2010). Pressure increments favor water infiltration within the protein nonpolar core, providing an opportunity to assess destabilized hydrated states (de Oliveira and Silva, 2015). Hydration effects in protein misfolding and amyloid conversion have been studied over the past 20 years in several macromolecular systems (Pedrote et al., 2018, Silva et al., 2018, Silva et al., 2010). Here, the “swollen states” captured for M237I-p53 are more hydrated than those formed by wt-p53, explaining mutant sensitivity to water infiltration and the loss of structural rigidity.

The p53 fold offers an opportunity to track conformational changes by reporting the fluorescence of its Tyr and single Trp residues. In the native state, the major signal comes from the Tyr residues, whereas the single Trp residue is quenched owing to the transfer of energy between Tyr residues within the excited state. Once p53 starts to unfold, Trp quenching is lost and Trp fluorescence becomes the major contribution to the signal (Bullock et al., 2000). Furthermore, the Tyr residues within p53C (eight in total) are spread throughout the entire domain, which offers an additional advantage to map overall hydration in this case. By reporting the fluorescence of these internal probes, we have shown the origins of the destabilization of mutant p53 in solution. Furthermore, these destabilized M237I-p53 states are preamyloidogenic species in solution, as revealed by the rapid ThT kinetic traces, negative staining microscopy, and increased protein hydrophobicity in the solvent. In cell-based approaches, M237I-p53 is strongly expressed in chemoresistant glioblastoma cells under ER stress and accumulates to the same level as the amyloid species.

Although M237I is not classified as a hotspot mutation, similarities in terms of thermodynamic stability, location, and ability to disrupt DNA binding between the M237I-p53 mutant and the R175H p53 structural mutant were previously reported (Bullock et al., 2000). Using molecular dynamic simulations, we observed regions within the DNA-binding domain of wt-p53 and the hotspot R175H-p53 mutant with an elevated incidence of exposed backbone hydrogen bonds (BHBs) compared with those of the p53 family members p63 and p73 (Cino et al., 2016). Protection of BHBs is an important feature of structural stability and the ability to form amyloid assemblies (Fernandez et al., 2003). Hydrophobic shielding of BHBs may reduce hydration by preventing the interaction of labile bonds with water molecules. In the case of M237I-p53, movement of the Ile side chain away from the Zn^2+^-binding motif may expose labile BHB sites for hydration. We did not investigate the effects of the M237I-p53 mutation on Zn^2+^ displacement, but the related R175H hotspot mutation accelerates the rate of Zn^2+^ loss (Butler and Loh, 2003). Notably, underprotected BHBs, also called dehydrons, act as sites for water attack, promote amyloid formation, and can be used as a strategy to target aggregation (Accordino et al., 2013, Wilcken et al., 2012). Recently, we have been pursuing different strategies to prevent p53 aggregation (Ferraz da Costa et al., 2018, Pedrote et al., 2018, Rangel et al., 2019, Silva et al., 2018). More importantly, the M237I mutation is fairly close to the previously identified underprotected BHB regions, reinforcing the role of hydration in p53 amyloid aggregation (Cino et al., 2016). We believe that this mutation disturbs the fragile wt-p53C structure by increasing hydration and protein hydrophobicity to the solvent. If increased hydration proves to be common to other destabilized p53 mutations, p53 dehydrons may be used as an alternative platform to avert the gain-of-function phenotype of oligomeric p53 compositions.

### Limitations of the Study

Caveats of this work include the absence of isogenic cell lines in our experiments and testing the chemoresistant p53 mutation in other cancer cells to directly attribute the chemoresistance phenotype to aggregated p53 species. Another drawback is that we are not able to certify whether the oligomeric states captured by the N&B analysis present the typical features of amyloids.

## Methods

All methods can be found in the accompanying Transparent Methods supplemental file.

## Supplemental Information

Document S1. Transparent Methods, Figures S1–S14, and Table S1

## References

[bib1] Accordino S.R., Rodriguez Fris J.A., Appignanesi G.A. (2013). Wrapping effects within a proposed function-rescue strategy for the Y220C oncogenic mutation of protein p53. PLoS One.

[bib2] Alexandrova E.M., Yallowitz A.R., Li D., Xu S., Schulz R., Proia D.A., Lozano G., Dobbelstein M., Moll U.M. (2015). Improving survival by exploiting tumour dependence on stabilized mutant p53 for treatment. Nature.

[bib3] Ano Bom A.P., Rangel L.P., Costa D.C., de Oliveira G.A., Sanches D., Braga C.A., Gava L.M., Ramos C.H., Cepeda A.O., Stumbo A.C. (2012). Mutant p53 aggregates into prion-like amyloid oligomers and fibrils: implications for cancer. J. Biol. Chem..

[bib4] Barak Y., Juven T., Haffner R., Oren M. (1993). mdm2 expression is induced by wild type p53 activity. EMBO J..

[bib5] Bisio A., Ciribilli Y., Fronza G., Inga A., Monti P. (2014). TP53 mutants in the tower of babel of cancer progression. Hum. Mutat..

[bib6] Bom A.P., Freitas M.S., Moreira F.S., Ferraz D., Sanches D., Gomes A.M., Valente A.P., Cordeiro Y., Silva J.L. (2010). The p53 core domain is a molten globule at low pH: functional implications of a partially unfolded structure. J. Biol. Chem..

[bib7] Bullock A.N., Henckel J., DeDecker B.S., Johnson C.M., Nikolova P.V., Proctor M.R., Lane D.P., Fersht A.R. (1997). Thermodynamic stability of wild-type and mutant p53 core domain. Proc. Natl. Acad. Sci. U S A.

[bib8] Bullock A.N., Henckel J., Fersht A.R. (2000). Quantitative analysis of residual folding and DNA binding in mutant p53 core domain: definition of mutant states for rescue in cancer therapy. Oncogene.

[bib9] Butler J.S., Loh S.N. (2003). Structure, function, and aggregation of the zinc-free form of the p53 DNA binding domain. Biochemistry.

[bib10] Cino E.A., Soares I.N., Pedrote M.M., de Oliveira G.A., Silva J.L. (2016). Aggregation tendencies in the p53 family are modulated by backbone hydrogen bonds. Sci. Rep..

[bib11] Cordeiro Y., Kraineva J., Ravindra R., Lima L.M., Gomes M.P., Foguel D., Winter R., Silva J.L. (2004). Hydration and packing effects on prion folding and beta-sheet conversion. High pressure spectroscopy and pressure perturbation calorimetry studies. J. Biol. Chem..

[bib12] de Oliveira G.A., Silva J.L. (2015). A hypothesis to reconcile the physical and chemical unfolding of proteins. Proc. Natl. Acad. Sci. U S A.

[bib13] Digman M.A., Dalal R., Horwitz A.F., Gratton E. (2008). Mapping the number of molecules and brightness in the laser scanning microscope. Biophys. J..

[bib14] Digman M.A., Stakic M., Gratton E. (2013). Raster image correlation spectroscopy and number and brightness analysis. Methods Enzymol..

[bib15] Dumaz N., Meek D.W. (1999). Serine15 phosphorylation stimulates p53 transactivation but does not directly influence interaction with HDM2. EMBO J..

[bib16] Fernandez A., Kardos J., Scott L.R., Goto Y., Berry R.S. (2003). Structural defects and the diagnosis of amyloidogenic propensity. Proc. Natl. Acad. Sci. U S A.

[bib17] Ferrão-Gonzales A.D., Palmieri L., Valory M., Silva J.L., Lashuel H., Kelly J.W., Foguel D. (2003). Hydration and packing are crucial to amyloidogenesis as revealed by pressure studies on transthyretin variants that either protect or worsen amyloid disease. J. Mol. Biol..

[bib18] Ferraz da Costa D.C., Campos N.P.C., Santos R.A., Guedes-da-Silva F.H., Martins-Dinis M., Zanphorlin L., Ramos C., Rangel L.P., Silva J.L. (2018). Resveratrol prevents p53 aggregation in vitro and in breast cancer cells. Oncotarget.

[bib19] Finlay C.A., Hinds P.W., Tan T.H., Eliyahu D., Oren M., Levine A.J. (1988). Activating mutations for transformation by p53 produce a gene product that forms an hsc70-p53 complex with an altered half-life. Mol. Cell Biol..

[bib20] Fischer N.W., Prodeus A., Malkin D., Gariepy J. (2016). p53 oligomerization status modulates cell fate decisions between growth, arrest and apoptosis. Cell Cycle.

[bib21] Forget K.J., Tremblay G., Roucou X. (2013). p53 Aggregates penetrate cells and induce the co-aggregation of intracellular p53. PLoS One.

[bib22] Furnari F.B., Fenton T., Bachoo R.M., Mukasa A., Stommel J.M., Stegh A., Hahn W.C., Ligon K.L., Louis D.N., Brennan C. (2007). Malignant astrocytic glioma: genetics, biology, and paths to treatment. Genes Dev..

[bib23] Gaglia G., Guan Y., Shah J.V., Lahav G. (2013). Activation and control of p53 tetramerization in individual living cells. Proc. Natl. Acad. Sci. U S A.

[bib24] Ghosh S., Salot S., Sengupta S., Navalkar A., Ghosh D., Jacob R., Das S., Kumar R., Jha N.N., Sahay S. (2017). p53 amyloid formation leading to its loss of function: implications in cancer pathogenesis. Cell Death Differ..

[bib25] Green D.R., Kroemer G. (2009). Cytoplasmic functions of the tumour suppressor p53. Nature.

[bib26] Ham S.W., Jeon H.Y., Jin X., Kim E.J., Kim J.K., Shin Y.J., Lee Y., Kim S.H., Lee S.Y., Seo S. (2019). TP53 gain-of-function mutation promotes inflammation in glioblastoma. Cell Death Differ..

[bib27] Hetz C. (2012). The unfolded protein response: controlling cell fate decisions under ER stress and beyond. Nat. Rev. Mol. Cell Biol..

[bib28] Hetz C., Martinon F., Rodriguez D., Glimcher L.H. (2011). The unfolded protein response: integrating stress signals through the stress sensor IRE1alpha. Physiol. Rev..

[bib29] Ishimaru D., Andrade L.R., Teixeira L.S., Quesado P.A., Maiolino L.M., Lopez P.M., Cordeiro Y., Costa L.T., Heckl W.M., Weissmuller G. (2003). Fibrillar aggregates of the tumor suppressor p53 core domain. Biochemistry.

[bib30] Ishimaru D., Maia L.F., Maiolino L.M., Quesado P.A., Lopez P.C.M., Almeida F.C.L., Valente A.P., Silva J.L. (2003). Conversion of wild-type p53 core domain into a conformation that mimics a hot-spot mutant. J. Mol. Biol..

[bib31] Ishimaru D., Ano Bom A.P., Lima L.M., Quesado P.A., Oyama M.F., de Moura Gallo C.V., Cordeiro Y., Silva J.L. (2009). Cognate DNA stabilizes the tumor suppressor p53 and prevents misfolding and aggregation. Biochemistry.

[bib32] Katz C., Low-Calle A.M., Choe J.H., Laptenko O., Tong D., Joseph-Chowdhury J.N., Garofalo F., Zhu Y., Friedler A., Prives C. (2018). Wild-type and cancer-related p53 proteins are preferentially degraded by MDM2 as dimers rather than tetramers. Genes Dev..

[bib33] King F.W., Wawrzynow A., Hohfeld J., Zylicz M. (2001). Co-chaperones Bag-1, Hop and Hsp40 regulate Hsc70 and Hsp90 interactions with wild-type or mutant p53. EMBO J..

[bib34] Kluth M., Harasimowicz S., Burkhardt L., Grupp K., Krohn A., Prien K., Gjoni J., Hass T., Galal R., Graefen M. (2014). Clinical significance of different types of p53 gene alteration in surgically treated prostate cancer. Int. J. Cancer.

[bib35] Kovachev P.S., Banerjee D., Rangel L.P., Eriksson J., Pedrote M.M., Martins-Dinis M., Edwards K., Cordeiro Y., Silva J.L., Sanyal S. (2017). Distinct modulatory role of RNA in the aggregation of the tumor suppressor protein p53 core domain. J. Biol. Chem..

[bib36] Lasagna-Reeves C.A., Clos A.L., Castillo-Carranza D., Sengupta U., Guerrero-Munoz M., Kelly B., Wagner R., Kayed R. (2013). Dual role of p53 amyloid formation in cancer; loss of function and gain of toxicity. Biochem. Biophys. Res. Commun..

[bib37] Lee S.H., Lee S.J., Chung J.Y., Jung Y.S., Choi S.Y., Hwang S.H., Choi D., Ha N.C., Park B.J. (2009). p53, secreted by K-Ras-Snail pathway, is endocytosed by K-Ras-mutated cells; implication of target-specific drug delivery and early diagnostic marker. Oncogene.

[bib38] Lee S.H., Woo T.G., Lee S.J., Kim J.S., Ha N.C., Park B.J. (2013). Extracellular p53 fragment re-enters K-Ras mutated cells through the caveolin-1 dependent early endosomal system. Oncotarget.

[bib39] Levy C.B., Stumbo A.C., Ano Bom A.P., Portari E.A., Cordeiro Y., Silva J.L., De Moura-Gallo C.V. (2011). Co-localization of mutant p53 and amyloid-like protein aggregates in breast tumors. Int. J. Biochem. Cell Biol..

[bib40] Li Y., Guessous F., Kwon S., Kumar M., Ibidapo O., Fuller L., Johnson E., Lal B., Hussaini I., Bao Y. (2008). PTEN has tumor-promoting properties in the setting of gain-of-function p53 mutations. Cancer Res..

[bib41] Marutani M., Tonoki H., Tada M., Takahashi M., Kashiwazaki H., Hida Y., Hamada J., Asaka M., Moriuchi T. (1999). Dominant-negative mutations of the tumor suppressor p53 relating to early onset of glioblastoma multiforme. Cancer Res..

[bib42] Milinkovic V., Bankovic J., Rakic M., Milosevic N., Stankovic T., Jokovic M., Milosevic Z., Skender-Gazibara M., Podolski-Renic A., Pesic M. (2012). Genomic instability and p53 alterations in patients with malignant glioma. Exp. Mol. Pathol..

[bib43] Moll U.M., Petrenko O. (2003). The MDM2-p53 interaction. Mol. Cancer Res..

[bib44] Montes de Oca Luna R., Wagner D.S., Lozano G. (1995). Rescue of early embryonic lethality in mdm2-deficient mice by deletion of p53. Nature.

[bib45] Muller P.A., Vousden K.H. (2013). p53 mutations in cancer. Nat. Cell Biol..

[bib46] Nagpal J., Jamoona A., Gulati N.D., Mohan A., Braun A., Murali R., Jhanwar-Uniyal M. (2006). Revisiting the role of p53 in primary and secondary glioblastomas. Anticancer Res..

[bib47] Ohgaki H., Kleihues P. (2007). Genetic pathways to primary and secondary glioblastoma. Am. J. Pathol..

[bib48] Park S.J., Borin B.N., Martinez-Yamout M.A., Dyson H.J. (2011). The client protein p53 adopts a molten globule-like state in the presence of Hsp90. Nat. Struct. Mol. Biol..

[bib49] Pedrote M.M., de Oliveira G.A.P., Felix A.L., Mota M.F., Marques M.A., Soares I.N., Iqbal A., Norberto D.R., Gomes A.M.O., Gratton E. (2018). Aggregation-primed molten globule conformers of the p53 core domain provide potential tools for studying p53C aggregation in cancer. J. Biol. Chem..

[bib50] Rangel L.P., Ferretti G.D.S., Costa C.L., Andrade S., Carvalho R.S., Costa D.C.F., Silva J.L. (2019). p53 reactivation with induction of massive apoptosis-1 (PRIMA-1) inhibits amyloid aggregation of mutant p53 in cancer cells. J. Biol. Chem..

[bib51] Shieh S.Y., Ikeda M., Taya Y., Prives C. (1997). DNA damage-induced phosphorylation of p53 alleviates inhibition by MDM2. Cell.

[bib52] Silva J.L., Vieira T.C., Gomes M.P., Bom A.P., Lima L.M., Freitas M.S., Ishimaru D., Cordeiro Y., Foguel D. (2010). Ligand binding and hydration in protein misfolding: insights from studies of prion and p53 tumor suppressor proteins. Acc. Chem. Res..

[bib53] Silva J.L., De Moura Gallo C.V., Costa D.C., Rangel L.P. (2014). Prion-like aggregation of mutant p53 in cancer. Trends Biochem. Sci..

[bib54] Silva J.L., Oliveira A.C., Vieira T.C., de Oliveira G.A., Suarez M.C., Foguel D. (2014). High-pressure chemical biology and biotechnology. Chem. Rev..

[bib55] Silva J.L., Cino E.A., Soares I.N., Ferreira V.F., A P de Oliveira G. (2018). Targeting the prion-like aggregation of mutant p53 to combat cancer. Acc. Chem. Res..

[bib56] Soragni A., Janzen D.M., Johnson L.M., Lindgren A.G., Thai-Quynh Nguyen A., Tiourin E., Soriaga A.B., Lu J., Jiang L., Faull K.F. (2016). A designed inhibitor of p53 aggregation rescues p53 tumor suppression in ovarian carcinomas. Cancer Cell.

[bib57] Stommel J.M., Marchenko N.D., Jimenez G.S., Moll U.M., Hope T.J., Wahl G.M. (1999). A leucine-rich nuclear export signal in the p53 tetramerization domain: regulation of subcellular localization and p53 activity by NES masking. EMBO J..

[bib58] Vousden K.H., Lane D.P. (2007). p53 in health and disease. Nat. Rev. Mol. Cell Biol..

[bib59] Vyas P., Beno I., Xi Z., Stein Y., Golovenko D., Kessler N., Rotter V., Shakked Z., Haran T.E. (2017). Diverse p53/DNA binding modes expand the repertoire of p53 response elements. Proc. Natl. Acad. Sci. U S A.

[bib60] Walerych D., Kudla G., Gutkowska M., Wawrzynow B., Muller L., King F.W., Helwak A., Boros J., Zylicz A., Zylicz M. (2004). Hsp90 chaperones wild-type p53 tumor suppressor protein. J. Biol. Chem..

[bib61] Walter P., Ron D. (2011). The unfolded protein response: from stress pathway to homeostatic regulation. Science.

[bib62] Wang G., Fersht A.R. (2015). Propagation of aggregated p53: cross-reaction and coaggregation vs. seeding. Proc. Natl. Acad. Sci. U S A.

[bib63] Wang X., Chen J.X., Liu J.P., You C., Liu Y.H., Mao Q. (2014). Gain of function of mutant TP53 in glioblastoma: prognosis and response to temozolomide. Ann. Surg. Oncol..

[bib64] Wang X., Chen J.X., Liu Y.H., You C., Mao Q. (2013). Mutant TP53 enhances the resistance of glioblastoma cells to temozolomide by up-regulating O(6)-methylguanine DNA-methyltransferase. Neurol. Sci..

[bib65] Wang Y., Zhu S., Cloughesy T.F., Liau L.M., Mischel P.S. (2004). p53 disruption profoundly alters the response of human glioblastoma cells to DNA topoisomerase I inhibition. Oncogene.

[bib66] Whitesell L., Sutphin P., An W.G., Schulte T., Blagosklonny M.V., Neckers L. (1997). Geldanamycin-stimulated destabilization of mutated p53 is mediated by the proteasome in vivo. Oncogene.

[bib67] Whitesell L., Sutphin P.D., Pulcini E.J., Martinez J.D., Cook P.H. (1998). The physical association of multiple molecular chaperone proteins with mutant p53 is altered by geldanamycin, an hsp90-binding agent. Mol. Cell Biol..

[bib68] Wilcken R., Wang G., Boeckler F.M., Fersht A.R. (2012). Kinetic mechanism of p53 oncogenic mutant aggregation and its inhibition. Proc. Natl. Acad. Sci. U S A.

[bib69] Wu X., Bayle J.H., Olson D., Levine A.J. (1993). The p53-mdm-2 autoregulatory feedback loop. Genes Dev..

[bib70] Xia F., Liber H.L. (1997). The tumor suppressor p53 modifies mutational processes in a human lymphoblastoid cell line. Mutat. Res..

[bib71] Xu J., Reumers J., Couceiro J.R., De Smet F., Gallardo R., Rudyak S., Cornelis A., Rozenski J., Zwolinska A., Marine J.C. (2011). Gain of function of mutant p53 by coaggregation with multiple tumor suppressors. Nat. Chem. Biol..

[bib72] Yang-Hartwich Y., Soteras M.G., Lin Z.P., Holmberg J., Sumi N., Craveiro V., Liang M., Romanoff E., Bingham J., Garofalo F. (2015). p53 protein aggregation promotes platinum resistance in ovarian cancer. Oncogene.

[bib73] Zhao R., Davey M., Hsu Y.C., Kaplanek P., Tong A., Parsons A.B., Krogan N., Cagney G., Mai D., Greenblatt J. (2005). Navigating the chaperone network: an integrative map of physical and genetic interactions mediated by the hsp90 chaperone. Cell.

[bib74] Zhu J., Sammons M.A., Donahue G., Dou Z., Vedadi M., Getlik M., Barsyte-Lovejoy D., Al-awar R., Katona B.W., Shilatifard A. (2015). Gain-of-function p53 mutants co-opt chromatin pathways to drive cancer growth. Nature.

