# Supplementary material for: Oncogenic Gain of Function in Glioblastoma Is Linked to Mutant p53 Amyloid Oligomers
Source: iScience. 2020 Jan 8;23(2):100820. doi: 10.1016/j.isci.2020.100820 (PMC6976948; doi:10.1016/j.isci.2020.100820)
Supplement: Document S1. Transparent Methods, Figures S1–S14, and Table S1 [file mmc1.pdf]

iScience, Volume *23*

Supplemental Information

Oncogenic Gain of Function in Glioblastoma Is Linked to Mutant p53 Amyloid Oligomers

Murilo M. Pedrote, Michelle F. Motta, Giulia D.S. Ferretti, Douglas R. Norberto, Tania C.L.S. Spohr, Flavia R.S. Lima, Enrico Gratton, Jerson L. Silva, and Guilherme A.P. de Oliveira

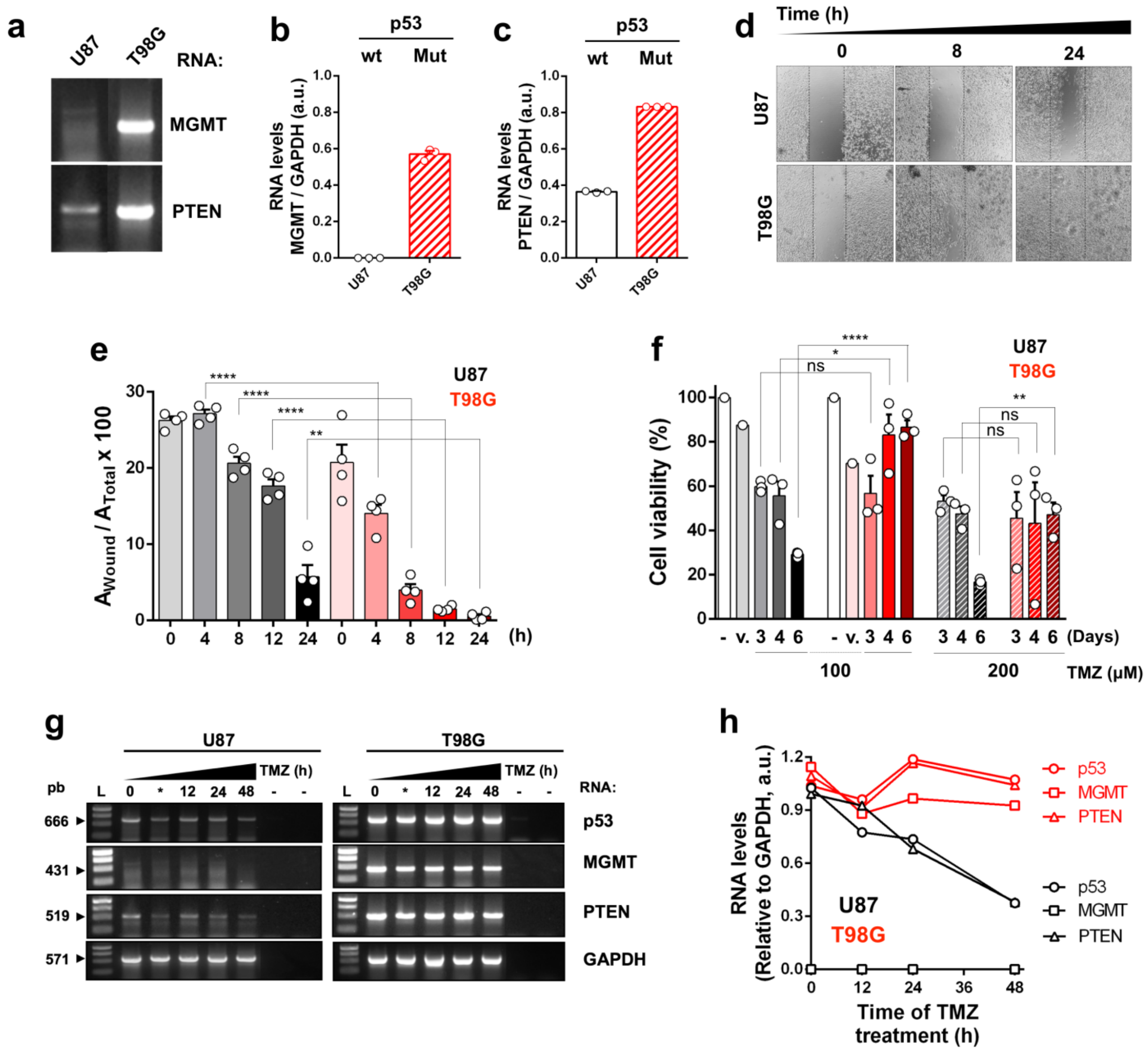

a
U87
T98G
RNA:
MGMT
PTEN
b
p53
wt
Mut
RNA levels
MGMT / GAPDH (a.u.)
U87
T98G
c
p53
wt
Mut
RNA levels
PTEN / GAPDH (a.u.)
U87
T98G
d
Time (h)
0
8
24
U87
T98G
e
U87
T98G
A_Wound / A_Total x 100
(h)
f
U87
T98G
Cell viability (%)
ns
(Days)
TMZ (μM)
g
U87
T98G
TMZ (h)
pb
L
RNA:
666
431
519
571
p53
MGMT
PTEN
GAPDH
h
RNA levels (Relative to GAPDH, a.u.)
U87
T98G
p53
MGMT
PTEN
Time of TMZ treatment (h)

Supplementary Figure 1. Chemoresistant features of glioblastoma derived cells, Related to Figure 1.

a, Agarose strips of wt p53-expressing (U87) and M237I p53-expressing (T98G) glioblastoma cells shows MGMT and PTEN transcript levels;

b, c, Dot plots showing MGMT and PTEN transcript levels, respectively, in wt p53-expressing (U87) and M237I p53-expressing (T98G) glioblastoma cells;

d, Bright-field images of U87 and T98G glioblastoma cells after 0, 8, and 24 h of scratch experiments;

e, Dot plot showing the migration rates following scratch experiments with wt p53- and M237I p53-expressing glioblastoma cells. The results are shown as the mean ± s.e.m. of (n = 4) area measurements from two independent scratch experiments (** P = 0.0024, **** P < 0.0001);

f, Dot plot showing the viability of wt p53- and M237I p53-expressing glioblastoma cells to temozolomide (TMZ) treatment. (-) Untreated cells; (v.) Mock condition. The results are shown as the mean ± s.e.m. of (n = 3) independent experiments (* P = 0.0199, ** P = 0.0081, **** P < 0.0001, and ns not significant);

g, Agarose strips of p53, MGMT, PTEN, and GAPDH of wt p53- (U87) and M237I p53-expressing (T98G) glioblastoma cells after 12, 24, and 48 h of TMZ treatment. (L) ladder; (*) Mock condition; (-) Negative controls;

h, Scatter plots showing the levels of p53, MGMT, and PTEN transcription in wt p53- (U87) and M237I p53-expressing (T98G) glioblastoma cells after 12, 24, and 48 h of TMZ treatment.

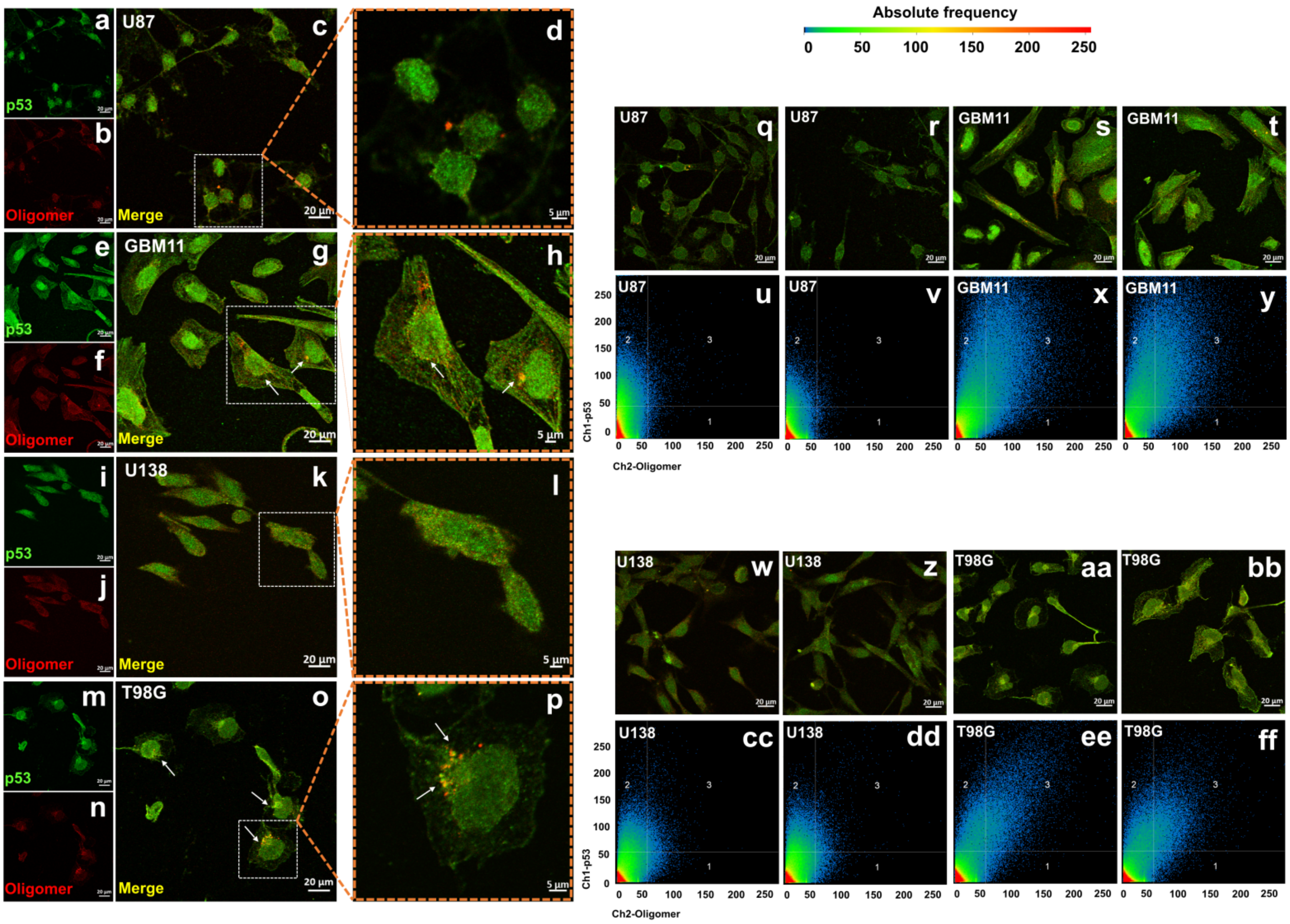

Supplementary Figure 2. Representative immunofluorescence assays and quantification, Related to Figure 1.

a-p, Immunofluorescence panels for *(a-d)* wt p53-expressing (U87), *(e-h)* wt p53-expressing (GBM11), *(i-l)* R273H p53-expressing (U138), and *(m-p)* M237I p53-expressing (T98G) glioblastoma cells showing p53 colocalization (white arrows) with amyloid oligomers. Scale bars, 20 µm; Insets, 5 µm;

q-ff, Immunofluorescence panels for *(q, r)* wt p53-expressing (U87), *(s, t)* wt p53-expressing (GBM11), *(w, z)* R273H p53-expressing (U138), and *(aa, bb)* M237I p53-expressing (T98G) glioblastoma cells showing merged p53 and amyloid oligomer channels used for pixel-by-pixel quantification. *(u-y)* and *(cc-ff)* show the pixel-by-pixel correlation plots between p53 and oligomer channels. Regions 1, 2, and 3 were classified as +/-, -/+, and +/+ (p53/oligomers). Pixel frequency is color-coded. Pixels within the double positive region 3 indicate colocalization between p53 and oligomers.

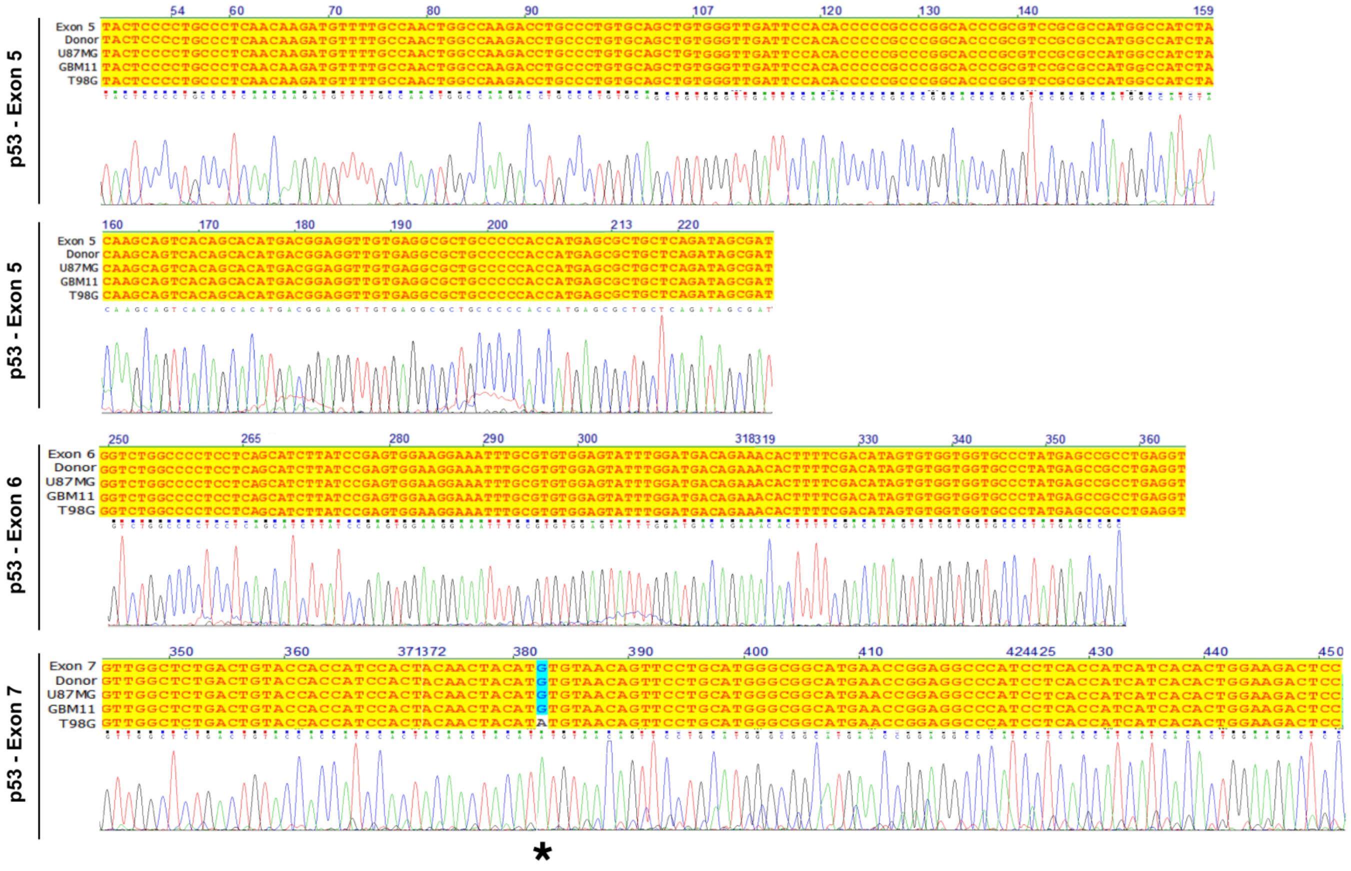

Supplementary Figure 3. DNA sequencing alignments of wt p53-expressing (U87 and GBM11) and M237I p53-expressing (T98G) glioblastoma cells showing the *TP53* gene region encoding the p53 DNA-binding domain. (*) ATG-to-ATA codon substitution leading to Met-to-Ile at position 237, Related to Figure 1.

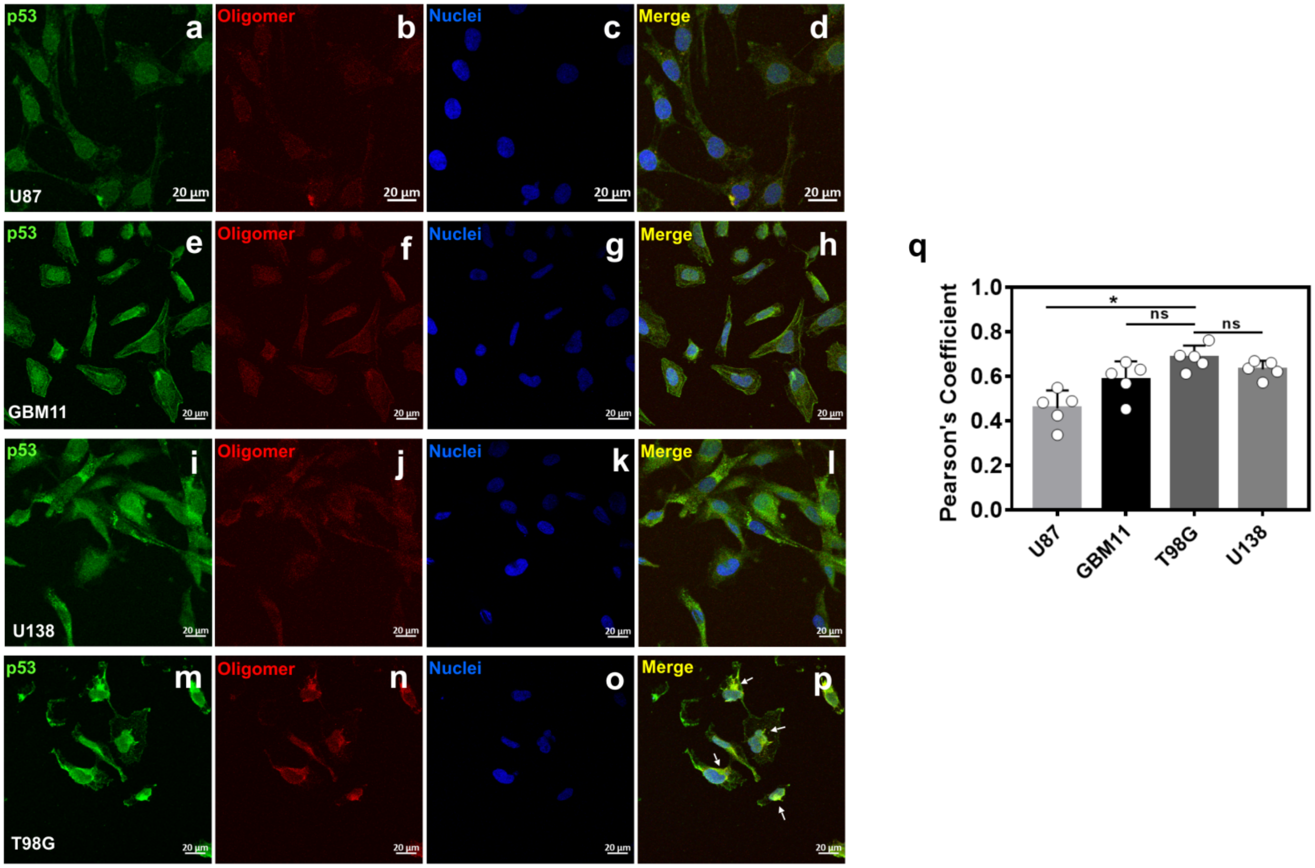

Supplementary Figure 4. Immunofluorescence quantification by Pearson's coefficient,

Related to Figure 1.

a-p, Immunofluorescence panels for *(a-d)* wt p53-expressing (U87), *(e-h)* wt p53-expressing (GBM11), *(i-l)* R273H p53-expressing (U138), and *(m-p)* M237I p53-expressing (T98G) glioblastoma cells showing p53 (green), amyloid oligomers (red), nuclei (blue), and merged channels (white arrows). Scale bars, 20 µm;

q, Dot plot showing Pearson's coefficient for p53 / oligomer colocalization of (n = 5) immunofluorescent images. (*) $0.01 < P < 0.05$; (ns) Not significant.

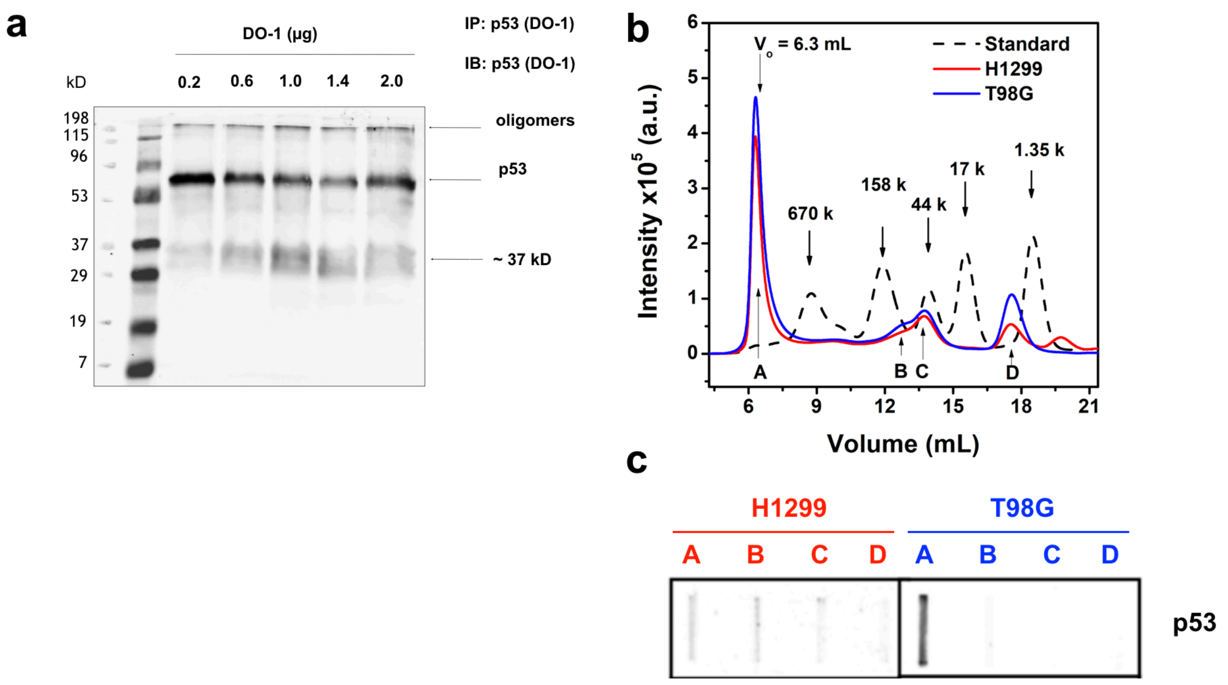

Supplementary Figure 5. Oligomeric species in M237I p53-expressing (T98G) glioblastoma cells, Related to Figure 1.

a, Immunoblot (IB) membrane showing fluorescent staining of p53 species after immunoprecipitation (IP) of p53 from M237I p53-expressing (T98G) glioblastoma whole cell extracts;

b, Line plot of the absorbance at 280 nm as a function of the elution volume showing the size exclusion pattern of M237I p53-expressing (T98G) glioblastoma and null-p53 H1299 carcinoma whole cell extracts. (V_o) Void volume; (k) kiloDaltons;

c, Dot blots showing p53 fluorescent staining of labeled (A-D) fractions in *(b)*.

Experiments were performed twice with similar results.

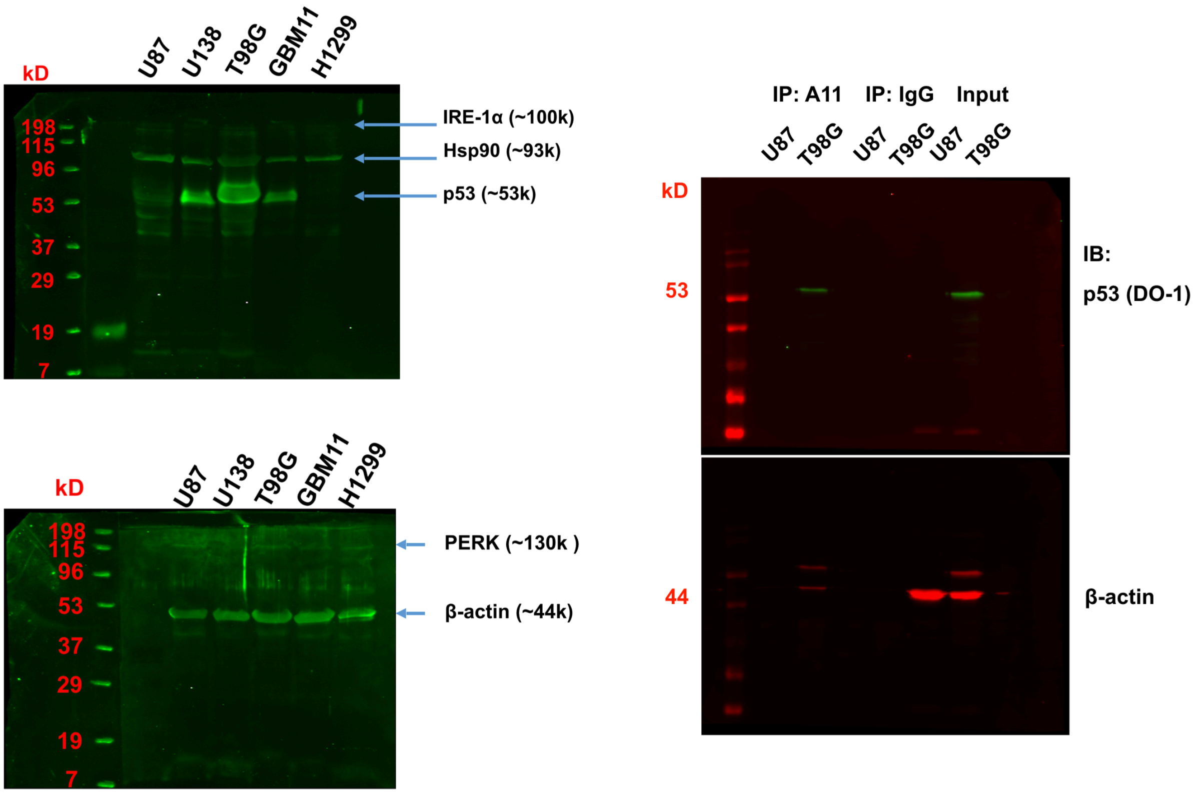

Supplementary Figure 6. Full immunoblot membranes showing proteins detected in this work, Related to Figure 2.

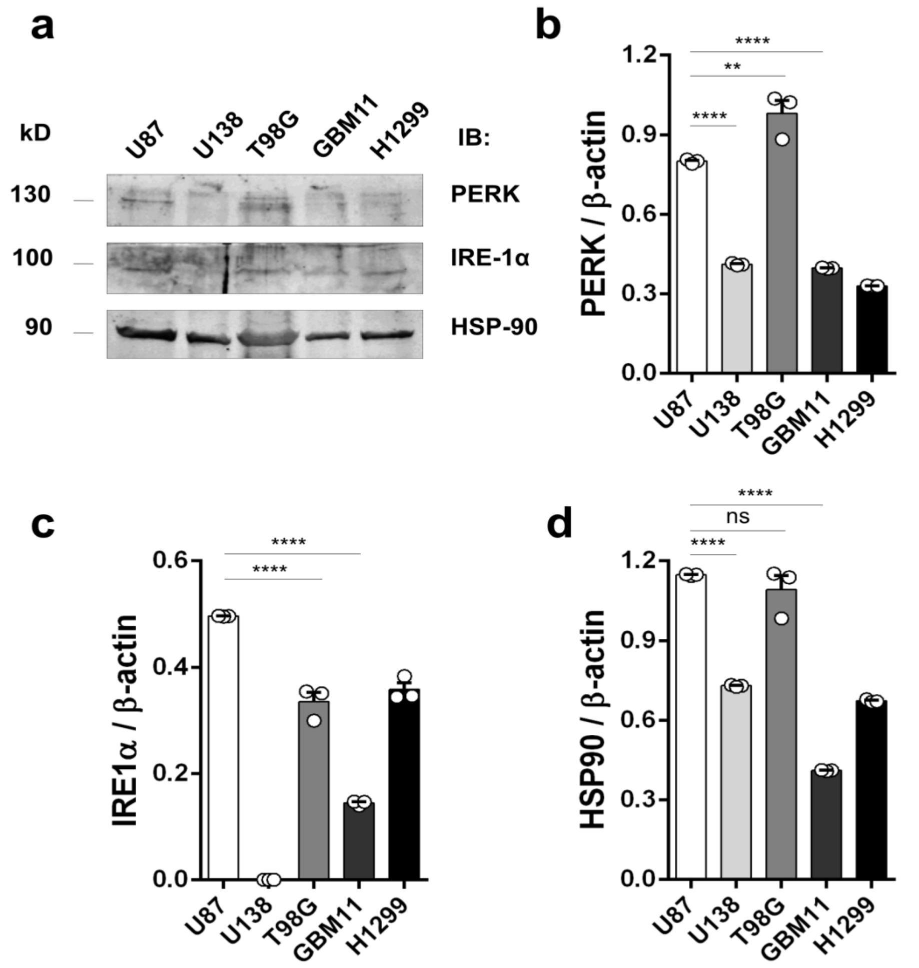

Supplementary Figure 7. Immunoblots for UPR sensors, Related to Figure 2.

a, Immunoblot (IB) strips showing the protein levels of inositol-requiring protein-1α (IRE-1α), protein kinase RNA-like ER kinase (PERK), and heat shock protein 90 (Hsp90) in glioblastoma and H1299 cells; **b-d,** Dot plots showing densitometry values obtained after normalizing PERK, IRE-1α, and Hsp90 levels to those of β-actin, respectively. The results are shown as the mean ± s.e.m. of (n = 3) densitometry measurements. Experiments were performed twice with similar results (**** $P < 0.0001$, ** $P = 0.0018$, and ns not significant).

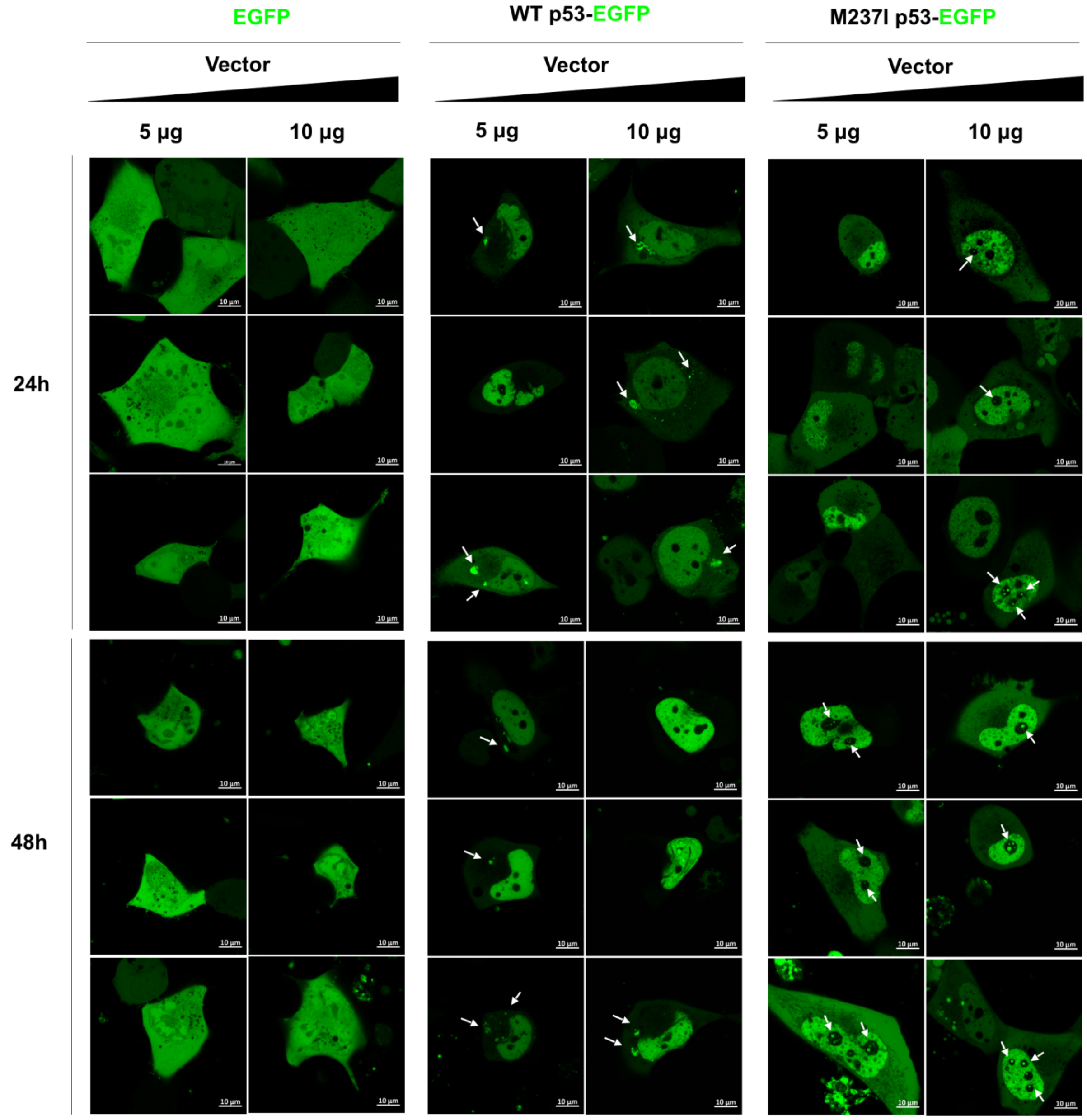

Supplementary Figure 8. Confocal images of EGFP-, wt p53-EGFP- and M237I p53-EGFP-transfected H1299 cells showing p53 puncta distribution (white arrows) within cytosolic and nucleolar compartments, Related to Figure 3. Scale bars, 10 µm;

Full-length wt p53-EGFP

a

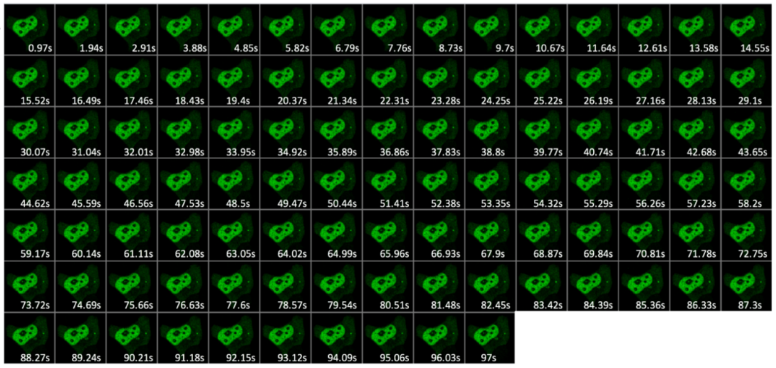

Full-length M237I p53-EGFP

b

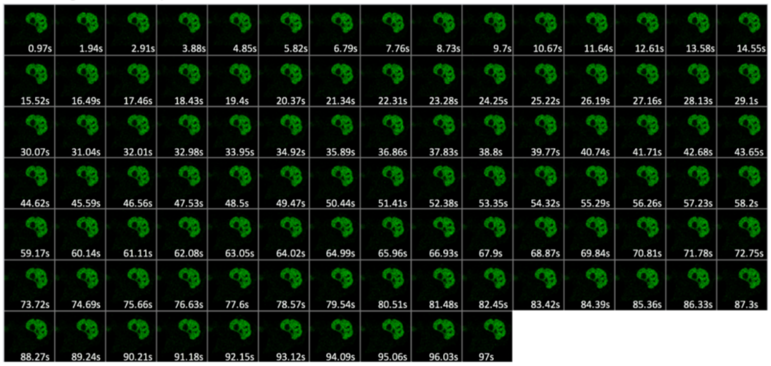

c

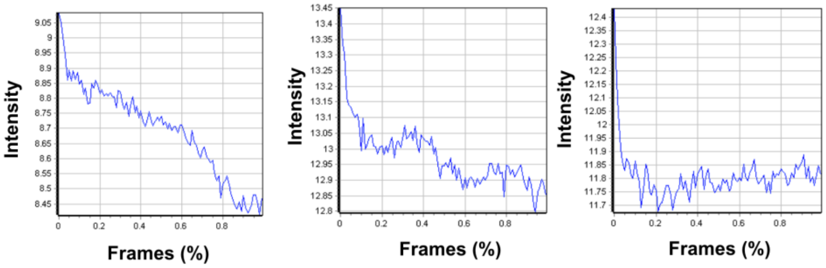

d

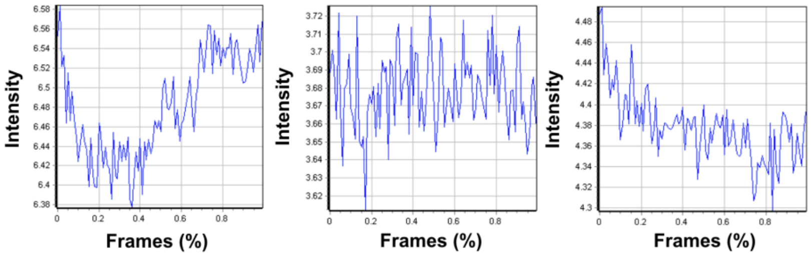

Supplementary Figure 9. N&B experimental settings, Related to Figure 3.

a, b, Representative frames for *(a)* wt p53-EGFP- and *(b)* M237I p53-EGFP-transfected H1299 imaged cell used as input for statistical number and brightness (N&B) analysis;

c, d, Representative line plots of the fluorescence intensity as a function of frames for *(c)* wt p53-EGFP- and *(d)* M237I p53-EGFP-transfected H1299 imaged cell showing low photobleaching contribution (less than 10%) through acquired frames.

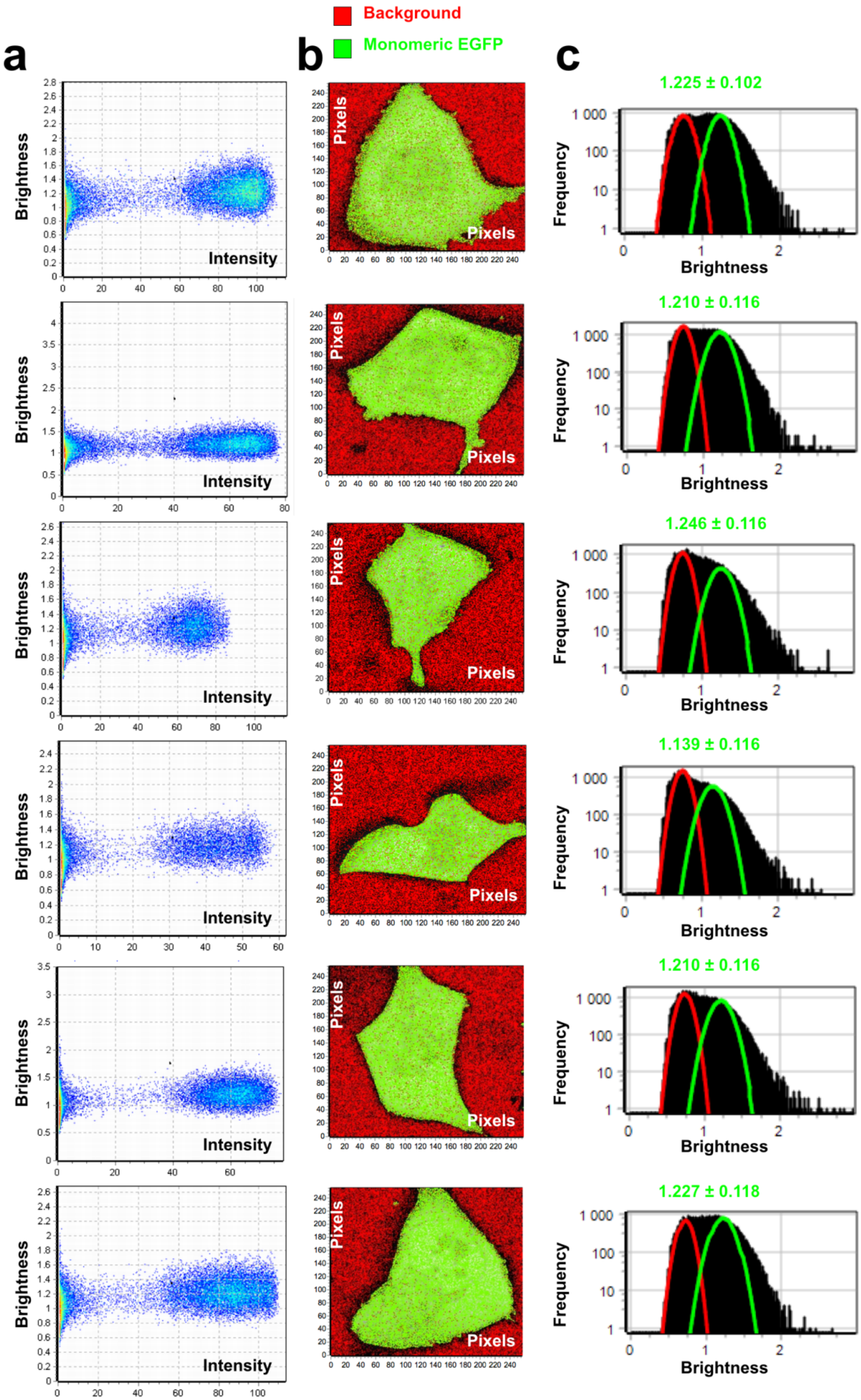

a
b
c
Background
Monomeric EGFP
Brightness
Intensity
Pixels
Frequency
1.225 ± 0.102
1.210 ± 0.116
1.246 ± 0.116
1.139 ± 0.116
1.210 ± 0.116
1.227 ± 0.118

Supplementary Figure 10. EGFP brightness normalization, Related to Figure 3.

a, Representative brightness plots of (n = 7) EGFP-transfected cells used to normalize brightness levels of monomeric EGFP;

b, Pixels corresponding to the immobile fraction i.e., background (red) and those from the EGFP protein (green) were selected from Gaussians of brightness distributions;

c, Brightness distributions of studied cells. Fitted gaussians (red and green) were used to depict pixel distributions in imaged cells *(b)*.

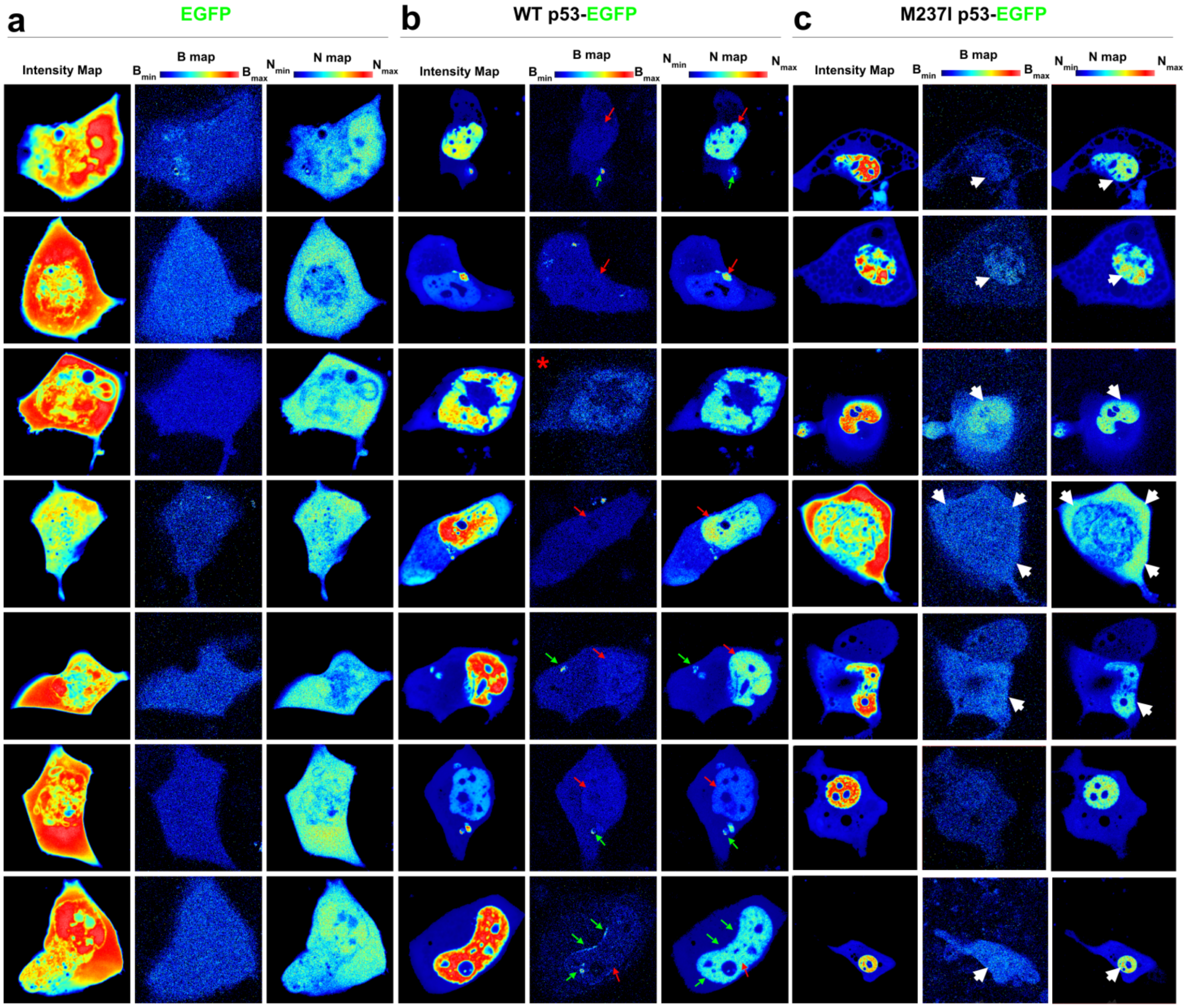

Supplementary Figure 11. Intensity, brightness, and number of molecule maps, Related to Figure 3.

a-c, Intensity, brightness, and number of molecule maps of *(a)* EGFP-, *(b)* wt p53-EGFP-, and *(c)* M237I p53-EGFP-transfected H1299 cells. One imaged wt p53-transfected cell exhibited equally brighter pixels within the nuclear region when compared to M237I p53-transfected cells (red asterisk). Red arrows show increased number of wt p53 molecules not predominantly overlapping with bright pixel regions; Green and white arrows show wt- and M237I-p53 containing increased number of molecules overlapping with brighter pixels, respectively.

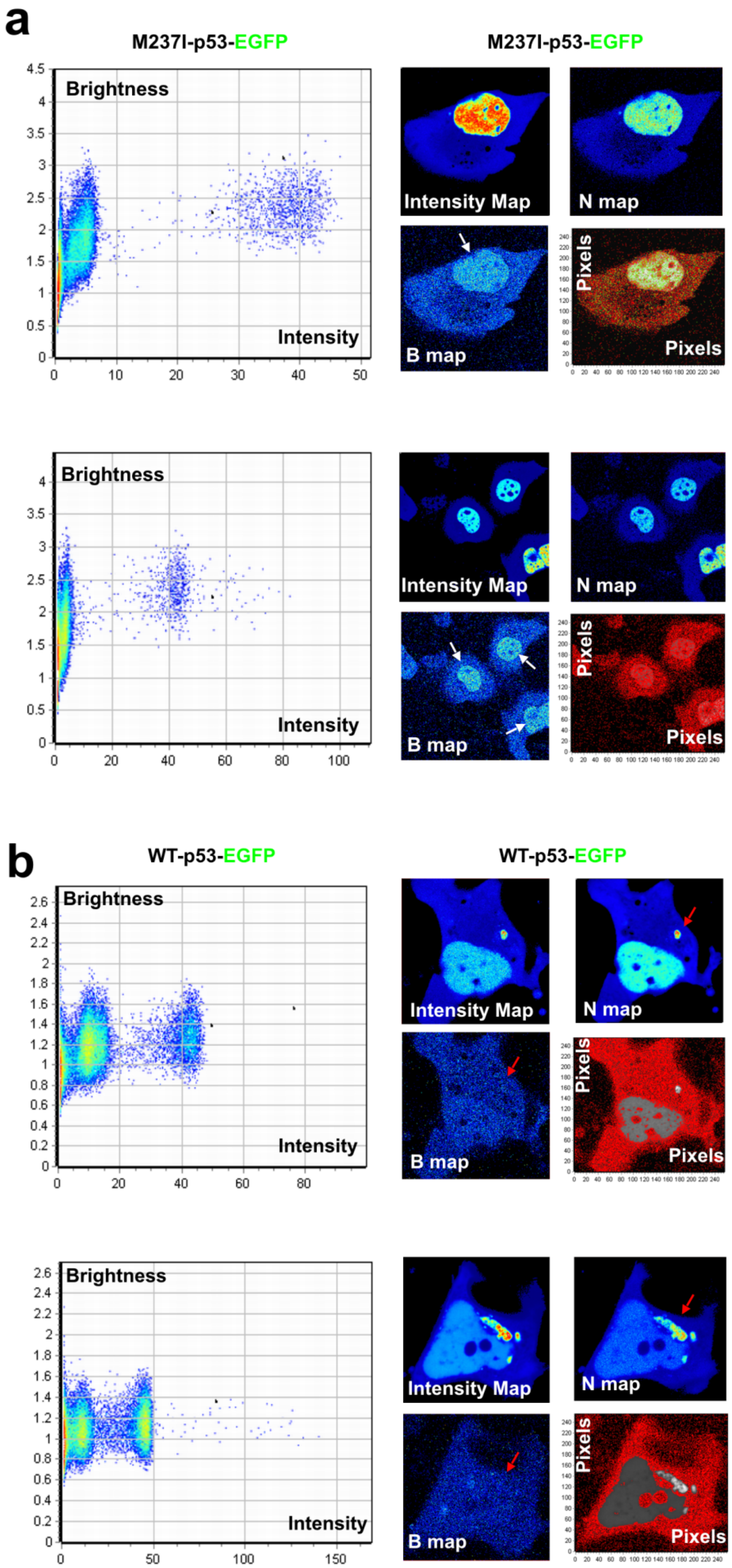
a
M237I-p53-EGFP
M237I-p53-EGFP
Brightness
Intensity
Intensity Map
N map
B map
Pixels
Pixels
Brightness
Intensity
Intensity Map
N map
B map
Pixels
Pixels
b
WT-p53-EGFP
WT-p53-EGFP
Brightness
Intensity
Intensity Map
N map
B map
Pixels
Pixels
Brightness
Intensity
Intensity Map
N map
B map
Pixels
Pixels

Supplementary Figure 12. WT- and M237I p53-EGFP brightness distributions, Related to Figure 4.

a, b, Representative cells showing *(a)* M237I p53-EGFP and *(b)* wt p53-EGFP brightness plots, intensity, B, and N maps. Arrows colored white and red show the same as in Supplementary Figure 11.

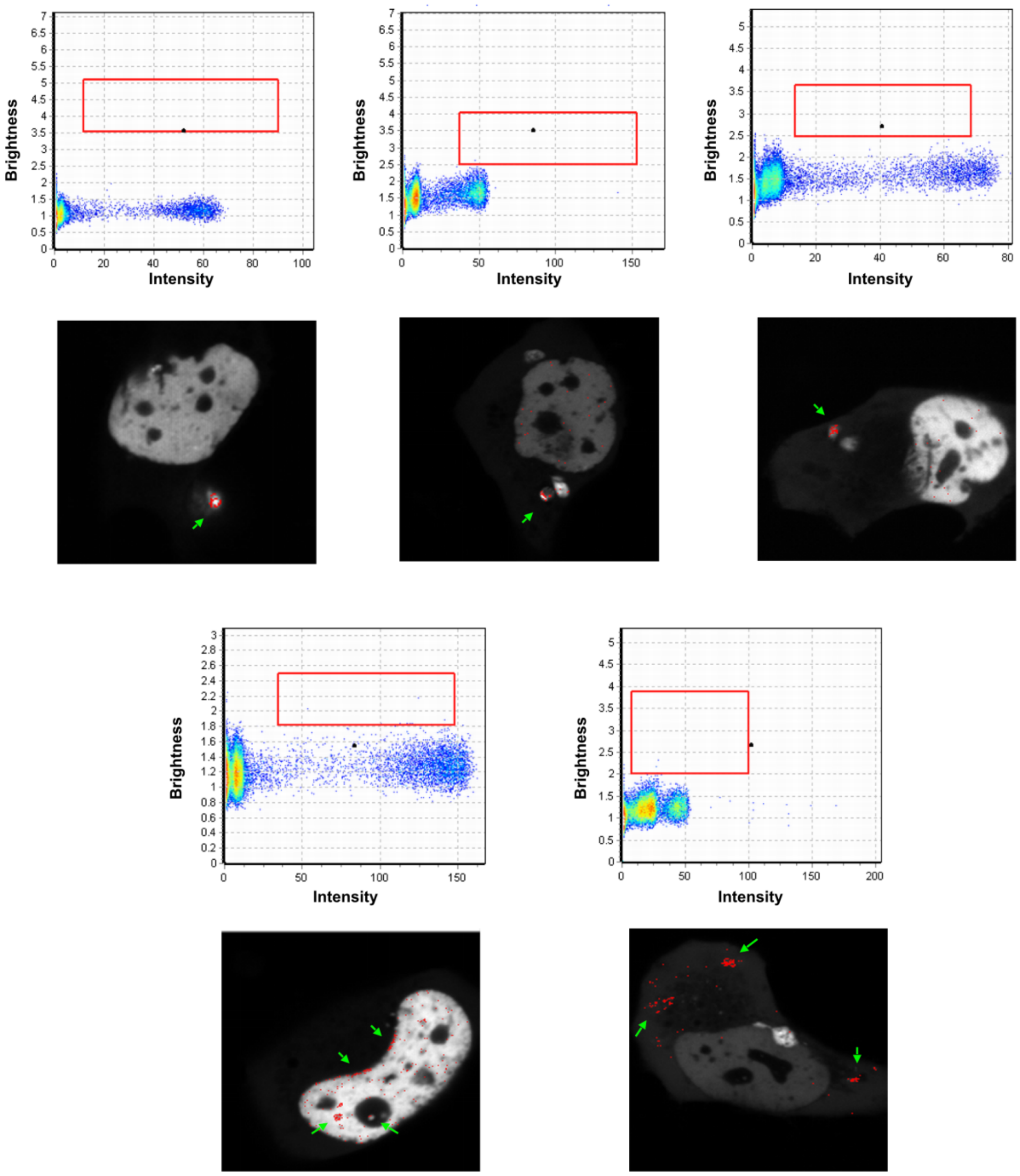

Supplementary Figure 13. Higher-order oligomeric states captured by N&B analysis, Related to Figure 4. Representative brightness plots of wt p53-EGFP-transfected H1299 cells showing pixels with higher B values (red rectangles) and their distribution within the cells (green arrows).

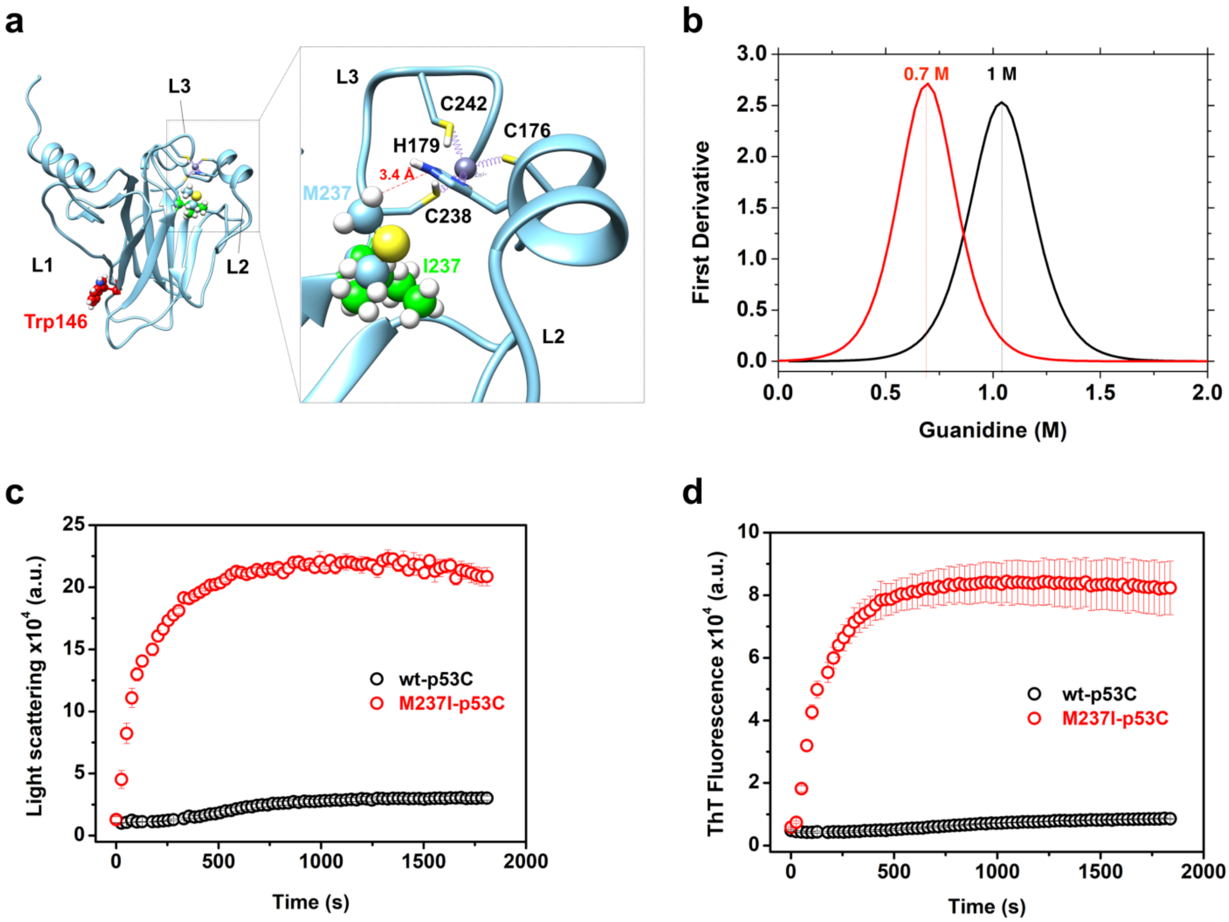

Supplementary Figure 14. Wt- and M237I p53 DNA-binding domain (p53C) behavior in solution, Related to Figures 5 and 6.

a, Ribbon representation of the p53C structure (PDB 2FEJ). L1-L3 stands for loop regions. Zoomed-in image shows the Zn^{2+}-binding site. Residues coordinating the Zn^{2+} ion are highlighted as sticks. Met-to-Ile substitution at position 237 is shown as spheres to emphasize side chain dislocation;

b, Line graph showing first derivative of the chemical-induced wt- and M237I p53C unfolding;

c, d, Kinetic traces showing *(c)* light scattering and *(d)* thioflavin T binding to wt- and M237I p53C.

Transparent Methods

Cell culture. The human glioblastoma cell lines used in this study comprised U87 (carrying wt-p53), T98G (carrying M237I-p53), U138 (carrying R273H-p53) (Cell lines were obtained from The Rio de Janeiro Cell Bank (BCRJ) and GBM11 (carrying wt-p53). The human H1299 non-small-cell lung carcinoma cell line was used as a model for null p53 (BCRJ). GBM11 cells were obtained by surgical biopsy from a 57-year-old male patient bearing a recurrent glioblastoma previously treated with TMZ concomitantly with radiotherapy, who had given written consent to the study. All procedures were in agreement with the Brazilian Ministry of Health Ethics Committee (CONEP no. 2340). GBM11, T98G, and U87 cells were cultured in DMEM/F12; U138 cells were cultured in DMEM, and H1299 cells were cultured in RPMI-1640 medium. All cells were supplemented with 10% fetal bovine serum and maintained at 37°C in a humidified atmosphere containing 5% CO_2. Cell lines were certified by STR genotype with their identity checked against the American Type Culture Collection (ATCC) STR profile.

DNA sequencing. Exons 5 to 8 of the TP53 gene corresponding to the p53 DNA-binding domain from U87, T98G, and GBM11 cells were amplified with the p53 primers described in Supplementary Table 1 and purified using 1 unit (U) of FastAP thermosensitive alkaline phosphatase and 5 U of exonuclease I as follows: 37°C for 30 min and 85°C for 15 min. Purified products were directly submitted to DNA sequencing using an Applied Biosystems (ABI 3130) instrument. Sequence analysis of wt-TP53 and TP53 with a codon substitution to generate a Met-

to-Ile mutation at position 237 in T98G cells (Supplementary Fig. 3) was performed using AlignX Invitrogen Corporation and Chromas Lite 2.1 software.

Wound-healing assay. Based upon the migration capacity of cells, we tested the motility of wt- and M237I-p53-expressing cells by a wound-healing assay (Liang et al., 2007). We cultured U87 and T98G cells on 12-well plates as described above. After washing the cells with phosphate-buffered saline (PBS, 140 mM NaCl, 2.7 mM KCl, 10 mM Na_2HPO_4, 1.8 mM KH_2PO_4), we scratched the monolayer with a 10 µL sterile plastic tip, and fresh media was added. We followed wound coverage at 0, 8, and 24 h after scratch by bright-field imaging. Quantification of cell recovery was determined using the following formula: wound area / total area × 100. Experiments were performed in duplicate.

Chemosensitivity assays. We cultured U87 and T98G cells in 24-well plates as described above. A TMZ stock (25 mM) was prepared in DMSO and stored at -80°C. TMZ solutions were directly diluted in fresh media to final concentrations of 100 and 200 µM (the final DMSO concentration was 0.008%). Cells were washed every day, and a new bolus of TMZ was added. Untreated cells were used as a control. We quantified cell viability by counting cells that excluded Tripan blue staining after 72, 96, and 144 h. Data are shown as the mean ± s.e.m. of several replicates.

Immunofluorescence. GBM cell lines (U87, T98G, U138 and GBM11) were grown on cover slips, washed twice with PBS, fixed in a formaldehyde solution (3.7%) and permeabilized with Triton X-100 (0.25%). Cells were then incubated with 5 mM ammonium chloride for 30 min and blocked for nonspecific antigenic sites for 1 h in 3% bovine serum albumin (BSA) (Sigma, #a7030) prepared in PBS. Next, cells were simultaneously labeled with a 1:200 dilution of mouse DO-1 monoclonal anti-p53 antibody (Santa Cruz, Inc. #sc-126) and a 1:1,000 dilution of A-11 anti-oligomer antibody (Millipore, #AB9234) overnight at 4°C in a humidified chamber. Cells were extensively washed with PBS and incubated with anti-mouse fluorescein isothiocyanate (FITC) 488 (Sigma, #F0257) and anti-rabbit IRDye® 680LT-conjugated (Li-Cor, #925-68023) secondary antibodies for 1 h at room temperature in a dark chamber. Finally, cells were washed with 3% BSA prepared in PBS, followed by extensive washes with PBS and fixed with Prolong® Gold Antifade Reagent with DAPI (Molecular Probes, #8961).

Confocal imaging. Immunofluorescence images were acquired with the LSM 510 Meta confocal laser scanning microscope (Carl Zeiss, Inc.) using excitation wavelengths of 488, 561 and 350 nm. For quantification analysis, ImageJ plugin JACoP (Just Another Colocalization Plugin) was used to analyze all immunofluorescence glioblastoma cell images, and Pearson coefficients were used to measure colocalization between p53 and oligomers. Five different images were used to calculate Pearson coefficients, which were plotted using GraphPad Prism 7. For fluorescence fluctuation spectroscopy, images were acquired with an ELYRA LSM 710 confocal laser scanning microscope (Carl Zeiss, Inc.) using an excitation wavelength of 488 nm and an EC Plan-Apochromat 63×/1.4 oil objective. During imaging, laser power was reduced to 0.2% to

avoid fluorophore bleaching (Supplementary Fig. 9). For N&B statistical analysis, we imaged a stack of 100 frames within an interval of approximately 2 min. Image sizes were 256 by 256 pixels, and the acquisition time was 12.61 μs/pixel. Cells from different preparations (n=10 cells for each studied condition) were imaged.

RT-PCR. A total of 7×10^{6} cells were washed with PBS, and RNA extraction was performed using the standard TRIzol protocol (Invitrogen, #15596026). After measuring the RNA concentration, 2 μg of total RNA was used for reverse transcription. The complementary DNA (cDNA) reaction mixture was composed of 0.8 μL 25 mM deoxynucleotide triphosphates (dNTPs) (Promega, #U1201), 2 μL 0.1 M dithiothreitol (DTT), 1 μL 100 pM random primer solution (Invitrogen, # 48190-011), 4 μL 5× buffer solution (Invitrogen), 1 μL 200 μg/μL Moloney murine leukemia virus reverse transcriptase (Promega, #M1701) and 1 μL 20U/μL RNAse inhibitor (Invitrogen, #AM2694). RNA solutions were heated at 65°C and placed on ice for 10 min, followed by incubation with the cDNA reaction mixture at 42°C for 40 min. Enzymes were inactivated at 65°C for 5 min, and cDNAs were stored at -20°C. Target primers are listed in Supplementary Table 1. PCR was carried out with 0.3 μL 5 U/μL Taq DNA polymerase (Phoneutria, #TAQPHT), 10× Buffer (Phoneutria, #TAQPHT), 1.5 mM $MgCl_2$, 0.15 mM dNTPs and 100 pM of each primer. Cycling conditions involved an initial denaturation step at 94°C for 5 min, followed by 30 cycles of 94°C for 30 sec + 57.5°C (for p53 and GAPDH), 55°C (for MGMT) or 58°C (for PTEN) for 1 min and 72°C for 1 min, with a final extension step at 72°C for 10 min. We visualized amplified products in 1% agarose gels stained with Gel Red

(Biotium, #41003), and densitometric analysis was performed with ImageJ software, v. 1.43r (National Institutes of Health, USA).

Immunoblots. To prepare protein extracts, cells were washed with PBS and lysed with RIPA buffer containing 150 mM sodium chloride, 1% NP-40, 0.5% deoxycholate, 0.1% sodium dodecyl sulfate, 50 mM Tris-Cl (pH 8.0) and 1× protease inhibitor cocktail (Sigma, #P8340). The protein concentration was determined as described by Lowry et al. (1951) using BSA (Sigma, #a7030) as a protein standard. One hundred micrograms of protein extracts were loaded onto SDS-PAGE gels (15%), transferred to polyvinylidene difluoride (PVDF) membranes (Uniscience, #926-31098) using the Semidry system (Bio-Rad, #170-3940), blocked for 1 h at 4°C in Odyssey® Blocking Buffer (Uniscience, #927-50000) and incubated overnight at 4°C with specific primary antibodies. The following antibodies and dilutions were used: anti-p53 (DO-1, Santa Cruz Biotechnology, Inc., sc-126), 1:200; anti-oligomer (A11, Millipore, #AB9234), 1:1,000; anti-GAPDH (Santa Cruz Biotechnology, Inc., #0411), 1:1,000; and anti-β-actin (Sigma, #A1978), 1:8,000. The membranes were washed 4 times with Tris-buffered saline, TBS (50 mM Tris-Cl, 150 mM NaCl, pH 7.5) and incubated for 1 h with IRDye® 800CW-labeled mouse (Uniscience, #926-32210) and rabbit (Uniscience, #926-32211) secondary antibodies diluted at 1:10,000. After 4 additional washes with TBS, visualization was carried out using the LI-COR Odyssey® scanner and software (LI-COR Biosciences). Densitometric quantification of bands was performed using ImageJ software, v. 1.43r (NIH, USA). For immunoprecipitation (IP) assays, we used Protein A/G Plus-agarose beads (Santa Cruz

Biotechnology Inc., #sc-2003) following manufacture instructions and 500 µg of whole U87 and T98G cell extracts.

Analytical size exclusion chromatography. Whole T98G and H1299 cell extracts were directly injected into a Superdex 200 10/300 GL (GE Lifesciences, #29-0915-96). All runs were performed in PBS (140 mM NaCl, 2.8 mM KCl, 10 mM Na_2HPO_4, and 1.8 mM KH_2PO_4, pH 7.4) at a flow rate of 0.7 mL min^{-1}, and the absorbance was monitored at 280 nm using a high performance liquid chromatograph system (Shimadzu). The column was previously calibrated using thyroglobulin, 670 kD; γglobulin, 158 kD; ovalbumin, 44 kD; myoglobin, 17 kD; and vitamin B12, 1.35 kD (Bio-Rad, #151-1901).

p53 protein preparations. To recombinantly express p53C constructs, the pET15b vector containing the core domain of wt- (Addgene, #24866) and M237I-p53 (introduced by site-directed mutagenesis) was transformed by heat shock into the BL21(DE3) *E. coli* strain, which was grown overnight at 37°C on LB agar with 100 µg/mL ampicillin. Cells were then grown with shaking at 180 rpm in LB to an $A_{600\ nm}$ of approximately 0.8 at 37°C, which was followed by T7 promoter induction with 1 mM isopropyl β-D-thiogalactoside; cells were then maintained at 25°C with shaking at 180 rpm for 2 h prior to a temperature decrease to 15°C overnight. Cells were harvested by centrifugation for 15 min at 10,000 × g and 4°C and stored at -20°C or resuspended in buffer A (50 mM Tris-Cl (pH 7.4) containing 150 mM NaCl, 5 mM DTT and one tablet of EDTA-free protease inhibitor cocktail (Sigma-Aldrich, 16424900)). Cells were sonicated, and soluble proteins were harvested through centrifugation at 18,000 × g for 15 min at

4°C. The supernatant was loaded onto Ni-NTA Superflow resin (Qiagen, #30450) previously equilibrated in buffer A using an Äkta Prime system at a flow rate of approximately 0.8-1 mL/ min. The column was then washed with buffer A until $A_{280\ nm}$ baseline stabilization, and weakly bound proteins were eluted with 5 column volumes of 5% buffer B (buffer A plus 0.5 M imidazole). p53 was eluted using a linear gradient of 5–100% buffer B. His-tag cleavage was performed using thrombin at a thrombin/protein ratio of 1:3,000 (w/w) for 2 h. Cleaved proteins were then loaded onto a Sephacryl 16/60 S-100 preparative column (GE Healthcare) equilibrated with buffer A as a final step to achieve high-purity p53C. The protein concentration was determined by $A_{280\ nm}$ using an extinction coefficient of 17,420 $M^{-1}.cm^{-1}$. Glycerol (5%) was added to p53 constructs before storage in liquid nitrogen.

LS measurements. LS was used to follow protein aggregation in pressure-induced measurements and kinetic experiments at physiological temperature. Samples were excited at 320 nm, the emission from 300 to 400 nm was recorded, and data were expressed as the area under the LS curve using an ISSK2 spectrofluorometer (ISS, Inc.). The sample concentration was 5 µM in 50 mM Tris-Cl (pH 7.4) containing 150 mM NaCl and 5 mM DTT. p53 samples were routinely centrifuged at 8,000 rpm 10 min prior to the experiments to eliminate any residual traces of aggregation.

ThT kinetics. ThT (25 µM) kinetics were recorded using 5 µM wt- or M237I-p53C in an ISSK2 spectrofluorometer (ISS, Inc.) upon excitation at 450 nm and emission at 471 nm under mild agitation. Reactions (total volume, 1 mL) were placed in a square cell with magnetic stirrers

(Hellma, #109004F-10-40) and temperature control (Quantum Northwest, #TC125) at 37°C throughout the assay. Experiments were performed three times with different protein preparations, and the results are expressed as the mean ± s.e.m. Mathematica (Wolfram) was used to calculate k_1 and k_2 rate constants according to the first-order equation below. Fitting of the data to the model can be obtained in the previously reported study (Pedrote et al., 2018).

$$F(t) = m\frac{\left(k_1 - k_2 + k_2 e^{-k_1 t} - k_1 e^{-k_2 t}\right)}{\left(k_1 - k_2\right) + k_3 t} + a,$$

where F is the intensity of ThT fluorescence as a function of time, t, and amplitude, m, is defined as the concentration of the monomeric protein multiplied by the raw fluorescence signal of ThT bound to the aggregate at the end of the kinetic experiment. The term k_3t is a small linear term related mainly to machine drift, and the parameter a is included to allow for a nonzero intercept at $t = 0$.

Transmission electron microscopy. Images of wt- and M237I-p53 were obtained after a 2 h incubation at 37°C or 2.68 kbar at 25°C. Samples 4 µL in volume were applied for 1 min to previously discharged carbon film on 200 mesh copper grids (EMS, #CF200-cu), gently dried with filter paper and stained for 5 sec with 2% uranyl acetate. Negatively stained images were acquired on a Philips Tecnai microscope operated at 80 kV at 21,000× and 46,000×.

Transfection.. Cells were plated on 35-mm dishes containing 14 mm glass coverslips (MatTek, #P35G-1.5-14-C), and transfection was performed using Lipofectamine 2000 reagent (Invitrogen) according to the protocol provided by the manufacturer. Cells transfected with 5 or 10 μg pEGFP-N1 vectors (Clontech) bearing the EGFP-tagged sequence at the C-terminus of the full-length wt-p53 sequence (Addgene, #11770) were imaged after 24, 48, and 72 h. For N&B experiments, cells were imaged after 24 h of transfection. The M237I-p53 mutation was introduced by site-directed mutagenesis using GenScript services and confirmed by DNA sequencing. The pEGFP-N1 null vector was used to express monomeric EGFP to standardize the brightness scale.

N&B statistical analysis. N&B applies pixel-by-pixel statistical analysis of the fluorescent intensity distribution within a stack of images. The average intensity ($\langle K \rangle$) can be obtained by measuring the fluorescent signal of each corresponding pixel in the stack, and according to the intensity fluctuations at each of these pixels, we know how broad the distribution, meaning the variance (σ^2), is. The larger the variance is, the fewer molecules there are that contribute to the average, while the brighter the molecule is. The apparent brightness (B) is then defined by the ratio of the variance over the average intensity ($\sigma^2/\langle K \rangle$); meanwhile, the apparent number of molecules (η) is related to the average intensity over the variance ($\langle K \rangle^2/\sigma^2$). The evaluation of image brightness maps (B_{maps}) allows the localization and quantification of oligomeric species in living cells. As σ^2 arises from contributions from *(i)* the molecular brightness (ε) of the number of molecules (η) fluctuating in and out of the excitation volume and *(ii)* the detector shot noise, B can be rewritten as $B = \varepsilon\eta + 1$. Because the immobile fraction (i.e., the background) has no

particle fluctuation and no σ^2 other than the shot noise, we attribute a value of 1 to the immobile contribution to B. After standardizing the B value of monomeric EGFP fluorophore (Supplementary Fig. 10) and assuming no changes in the quantum yield of EGFP upon oligomerization, we can determine the oligomerization status of EGFP-tagged proteins according to the number of fluorescent molecules within these aggregates from $B = \varepsilon\eta + 1$. We measured a B value of 1.2 for monomeric (η=1) EGFP. We calculated a B value of 1.4 for η=2 (dimers) and 1.8 for η=4 (tetramers); we can attribute any B values > 2 to η>4 (oligomers larger than tetramers). Few pixels with B values higher than 3 and lower than 4 were localized within puncta of wt-p53-transfected cells. We can attribute B values within this range to oligomeric states on the order of decamers (η=10) and 15-mer species. N&B analysis was performed using the SimFCS suite (www.lfd.uci.edu).

Pressure-induced spectroscopy. Experiments were performed using 5 µM wt- and M237I-p53C in 50 mM Tris-Cl (pH 7.4) containing 150 mM NaCl and 5 mM DTT. Fluorescence emission was acquired using an ISSK2 spectrofluorometer (ISS Inc.) equipped with a high-pressure optical cell (ISS Inc.). The cell contained three 10 mm-diameter sapphire windows placed at 90 degrees, allowing L-format acquisition of fluorescence measurements. Samples were excited at 280 nm to measure tyrosine and tryptophan probes, and emission was recorded from 290-400 nm at 2 nm increments. Ethanol was used as the hydrostatic liquid, and polypropylene tubing was used to seal pressure cuvettes, allowing efficient pressure transfer to the sample. Pressure cycles were carried out with increments of approximately 300-500 bars, followed by 5-10 min for system acclimation before emission spectra acquisition. The center of spectral mass (*v*) was used

according to the equation below to quantify changes in the fluorescence spectra at each pressure point. *Fi* stands for the emitted fluorescence at wavenumber λ.

$$v = \frac{\sum \lambda F_i}{\sum F_i}$$

Structural analysis. *In silico* site-directed mutagenesis analysis and structural evaluations were carried out in the UCSF Chimera suite (Pettersen et al., 2004). The Met-to-Ile substitution at position 237 of the p53 core domain was introduced into the NMR structure of the p53 core domain (PDB code 2FEJ) with the Dunbrack backbone-dependent rotamer library (Dunbrack, 2002). Clashes within 5 Å were minimized by the structure minimization tool implemented in Chimera. The structure was rendered by the Kyte-Doolittle hydrophobicity algorithm (Kyte and Doolittle, 1982), and maps were displayed as color-coded worms.

Statistical analysis and reproducibility. We used GraphPad Prism v.6 to perform all statistical tests. Based on experimental assays, we assumed normality and equal variances. We performed ordinary one-way ANOVA and Sidak's multiple comparisons tests assuming a single pooled variance. Correction of multiple comparisons were based on 95% CI. Values are expressed as the mean ± s.e.m. The results of statistical tests are indicated in each figure as * $0.01 < P < 0.05$; ** $0.001 < P < 0.01$; *** $0.0001 < P < 0.001$; and **** $P < 0.0001$.

All experiments were tested for reproducibility at least twice. Sample groups for each experiment were defined as follows: for immunofluorescence assays we used two wt-p53 (U87

and GBM11) and two mutant p53-expressing (U138 and T98G) cell lines to compare their p53 aggregation status; to show the chemoresistance phenotype of T98G cells, we used U87 cells for comparison as this cell line is a well-known glioblastoma standard, is easy to use, and expresses wt-p53. For size exclusion chromatography experiments, we used H1299 cells instead of U87 cells because the null p53 H1299 cell line is an ideal negative control of p53 species eluted within the void fraction. All previous glioblastoma cell lines were used for immunoblot experiments to assess the p53 expression levels. For N&B experiments we used H1299 cells for transfection as this cell line does not express endogenous p53, is easy to transfect, and can be easily examined by microscopy. Endogenous p53 contributes to the oligomerization status of transfected wt-p53 and M237I-p53.

Supplementary Table 1. Primers used for targets: p53, GAPDH, MGMT, and PTEN, Related to Figure 2

**p53**		
Primer	Sequence	Product (pb)
Forward	5’ GCT TCT TGC ATT CTG GGA CAG 3’	626
Reverse	5’ CTT CTT TGG CTG GGG AGA GG 3’	
**GAPDH**		
Primer	Sequence	Product (pb)
Forward	5’ ATC ACC ATC TTC CAG GAG GCG 3’	574
Reverse	5’ CCT GCT TCA CCA CCT TCT TG 3’	
**MGMT**		
Primer	Sequence	Product (pb)
Forward	5’ GCA GTA GGA TGG ATG TTT GA 3’	431
Reverse	5’ TAG GAA CTG CTG AGT GGA GA 3’	
**PTEN**		
Primer	Sequence	Product (pb)
Forward	5’ CAG AGA CAA AAA GGG AGT AAC TA 3’	519
Reverse	5’ GCT TTG TCT TTA TTT GCT TTG TC 3’	

References

Dunbrack, R.L., Jr. (2002). Rotamer libraries in the 21st century. Curr Opin Struct Biol *12*, 431-440.
Kyte, J., and Doolittle, R.F. (1982). A simple method for displaying the hydropathic character of a protein. J Mol Biol *157*, 105-132.
Liang, C.C., Park, A.Y., and Guan, J.L. (2007). In vitro scratch assay: a convenient and inexpensive method for analysis of cell migration in vitro. Nat Protoc *2*, 329-333.
Pettersen, E.F., Goddard, T.D., Huang, C.C., Couch, G.S., Greenblatt, D.M., Meng, E.C., and Ferrin, T.E. (2004). UCSF Chimera--a visualization system for exploratory research and analysis. J Comput Chem *25*, 1605-1612.
Pedrote, M.M., de Oliveira, G.A.P., Felix, A.L., Mota, M.F., Marques, M.A., Soares, I.N., Iqbal, A., Norberto, D.R., Gomes, A.M.O., Gratton, E., et al. (2018). Aggregation-primed molten globule conformers of the p53 core domain provide potential tools for studying p53C aggregation in cancer. J Biol Chem 293, 11374-11387.
